# Development and validation of a risk prediction model for 30-day readmission in elderly type 2 diabetes patients complicated with heart failure: a multicenter, retrospective study

**DOI:** 10.3389/fendo.2025.1534516

**Published:** 2025-02-27

**Authors:** Yuxin He, Yuan Yuan, Qingzhu Tan, Xiao Zhang, Yunyu Liu, Minglun Xiao

**Affiliations:** ^1^ Department of Medical Administration, Affiliated Banan Hospital of Chongqing Medical University, Chongqing, China; ^2^ Medical Recorods Department, Women and Children’s Hospital of Chongqing Medical University, Chongqing Health Center for Women and Children, Chongqing, China; ^3^ Medical Records and Statistics Room, Affiliated Banan Hospital of Chongqing Medical University, Chongqing, China; ^4^ Medical Insurance Department, Affiliated Banan Hospital of Chongqing Medical University, Chongqing, China; ^5^ Department of Gerontology, Affiliated Banan Hospital of Chongqing Medical University, Chongqing, China

**Keywords:** type 2 diabetes mellitus, heart failure, 30-day readmission, prediction model, electronic medical records

## Abstract

**Background:**

Elderly type 2 diabetes mellitus (T2DM) patients complicated with heart failure (HF) exhibit a high rate of 30-day readmission. Predictive models have been suggested as tools for identifying high-risk patients. Thus, we aimed to develop and validate a predictive model using multicenter electronic medical records (EMRs) data to estimate the risk of 30-day readmission in elderly T2DM patients complicated with HF.

**Methods:**

EMRs data of elderly T2DM patients complicated with HF from five tertiary hospitals, spanning 2012 to 2023, were utilized to develop and validate the 30-day readmission model. The model were evaluated using holdout data with the area under the receiver operating characteristic curve (AUROC), calibration curves, decision curve analysis (DCA), and clinical impact curves (CIC).

**Results:**

A total of 1899 patients were included, with 955, 409, and 535 in the derivation, internal validation, and external validation cohorts, respectively. Pulmonary infections (odds ratio [OR]: 3.816, 95% confidence interval [CI]: 2.377-6.128, *P* < 0.001), anti-hypertensive drug use (OR: 5.536, 95% CI: 1.658-18.486, *P* = 0.005), and neutrophil percentage-to-albumin ratio (NPAR) (OR: 1.144, 95% CI: 1.093-1.197, *P* < 0.001) were independent predictors of 30-day readmission risk. AUROC in the derivation, internal validation, and external validation cohorts were 0.782 (95% CI: 0.737-0.826), 0.746 (95% CI: 0.654-0.838), and 0.753 (95% CI: 0.684-0.813), respectively. The calibration curve, DCA results, and CIC results indicated that the model also possessed good predictive power. Additionally, an operation interface on a web page (https://cqykdxtjt.shinyapps.io/readmission/) was created for clinical practitioners to apply.

**Conclusion:**

A 30-day readmission risk prediction model was developed and externally validated. This model facilitates the targeting of interventions for elderly T2DM patients complicated with HF who are at high risk of an early readmission.

## 1 Introduction

There is a bidirectional link between type 2 diabetes mellitus (T2DM) and heart failure (HF), and their coexistence significantly increases the incidence and mortality rates of affected individuals (1). According to global records, about 537 million people worldwide suffer from diabetes, with over 90% being T2DM cases (2). T2DM leads to hemorheology due to abnormal glucose and lipid metabolism, thereby inducing and exacerbating HF. Compared to isolated HF, T2DM combined with HF results in more severe cardiac dysfunction, poorer prognosis, and higher expected mortality rate (3, 4). The study by Paolisso et al. also demonstrated that patients with hyperglycemia, as opposed to normoglycemia, exhibit higher levels of inflammatory markers and B-type natriuretic peptide, lower left ventricular ejection fraction, and increased rates of HF and mortality (5). Furthermore, the intersection of T2DM and HF primarily occurs in elderly patients, adding to the complexity of treatment (6).

Hospital readmission is a common adverse outcome in clinical practice, imposing various burdens on patients, such as increased medical costs and a heightened risk of mortality (7, 8). The 30-day readmission rate is an important indicator for assessing the quality of medical services (9). Salman et al.’s study demonstrated that HF readmission is associated with a higher mortality rate (4.9% vs 3.3%; *P* < 0.01) and an average cost increase of $13,000 per readmission over a 30-day period compared to non-HF readmission (10). Additionally, studies have shown that the 30-day readmission rates for patients with T2DM and chronic HF after discharge were as high as 21.0% and 19.8%, respectively (11, 12). In 2012, the Center for Medicare & Medicaid Services (CMS) developed the “Hospital Readmissions Reduction Program (HRRP)” to reduce readmission rates and improve medical quality by imposing financial penalties on hospitals with readmission rates higher than the national average (13). The goal of reducing readmission has sparked significant interest in researching and predicting hospital readmissions.

Most studies on patient readmission risk factors have focused on common diseases such as HF, acute myocardial infarction, and pneumonia (14). The degree of blood sugar control (15), the impact of hypoglycemia (16), and the presence or absence of HF (17) were common influencing factors for readmission in diabetes patients. Gek et al. analyzed data from the EMR system of hospitalized diabetic patients from 2008 to 2015 in Singapore, focusing on those who were readmitted within 30 days post-discharge (18). The results indicated that LOS was an independent risk factor for readmission, with a risk 2.22 times higher for LOS > 3 days compared to LOS ≤ 3 days. In a South Korean cohort study involving over 4,500 HF patients aged 40 and above, researchers developed a 30-day readmission risk prediction model incorporating 12 risk factors, achieving an AUROC of 0.710 (19). A survey in Australia utilized clinical data from patients aged 65 and above hospitalized for HF between 2003 and 2008 (20). Using a machine learning algorithm, they constructed a predictive model for 30-day readmission. Among a cohort of 10,757 HF patients, the 30-day readmission or mortality rate was 23.7%, and the model’s discriminability was 0.66. There is still a lack of studies evaluating the generalizability of prediction models across diverse populations, particularly in elderly patients with multiple comorbidities.

Therefore, this study aimed to further analyze and explore the influencing factors of 30-day readmission in elderly T2DM patients complicated with HF, and construct a readmission risk prediction model to achieve early risk screening for high-risk readmission patients. At the same time, the risk of readmission is visualized through nomogram, providing a simple assessment tool for clinical medical officers to take targeted intervention measures for high-risk patients, thereby improving adverse outcomes, reducing readmission rates, and alleviating the burden of public healthcare.

## 2 Methods

### 2.1 Study design and patients

This study adhered to the Transparent Reporting of a Multivariable Prediction Model for Individual Prognosis or Diagnosis (TRIPOD) guidelines (21). In this retrospective, multicenter study, electronic medical records (EMRs) data of 1,899 elderly T2DM patients complicated with HF were collected from five tertiary hospitals in southwest China between 2012 and 2023. A total of 889 patients were recruited from the Second Affiliated Hospital of Chongqing Medical University, 282 from the Third Affiliated Hospital of Chongqing Medical University, 114 from the University-Town Hospital of Chongqing Medical University, and 79 from Chongqing Southeast Hospital. Patients from these four centers were randomly divided (7:3) into a derivation cohort (n=955) and an internal validation cohort (n=409). This approach was chosen to maximize the generalizability of the model and to provide an unbiased evaluation of its performance on an independent dataset. Patients recruited from the Affiliated Banan Hospital of Chongqing Medical University were used as the external validation cohort (n=535).

This study was approved by the Ethics Committee of the Affiliated Banan Hospital of Chongqing Medical University (Ethical Approval No. BNLLKY2023037) and was conducted in accordance with the Declaration of Helsinki. Informed consent for participation was not required due to the retrospective design of the study, which was conducted in compliance with national legislation and institutional requirements.

### 2.2 Inclusion and exclusion criteria

To ensure scientific validity, internal consistency, clinical relevance, and data quality, we have established the following inclusion and exclusion criteria: the inclusion criteria are as follows: 1) age ≥ 65 years; 2) data obtained from 2012 to 2023; and 3) hospitalization(s) for T2DM complicated with HF. The exclusion criteria includes: 1) patients without a readmission record between 2012 and 2023 (n=3,217); 2) LOS < 2 days (n=16); 3) patients who died before discharge (n=8); 4) patients with end-stage liver diseases (chronic and acute liver failure, acute decompensation of liver cirrhosis, chronic liver failure, and hepatocellular carcinoma), end-stage renal disease (uremia), or diagnosed with malignant tumors (n=291), 5) patients complicated with severe mental illness or cognitive impairments (n=19); and 6) patients with missing data (n=245). After excluding patients who did not meet the selection criteria, a total of 5,695 patients were included in our study. Of these, 1,899 patients were included for predicting 30-day readmission. The selection process is illustrated in **Supplementary Figure S1**.

### 2.3 Outcome

The main outcome of this study was 30-day readmission. Readmission was defined to be a subsequent stay in the hospital for observation or an acute inpatient stay within 30 days after discharge, and excluding rehabilitation admissions, nursing home admissions, or scheduled admissions for surgeries or procedures. Hospital transfers were documented and treated as a single hospitalization episode if they occurred within the same admission period. Transfers between different admission periods were classified as separate readmissions. This approach was chosen to avoid double-counting hospitalizations and to ensure accurate measurement of the primary outcome.

### 2.4 Data collection

During the initial phase of clinical data analysis modeling, there are typically hundreds of feature variables, but only a few are truly relevant to the study’s target variable. Accurately incorporating the relevant feature variables in the analysis can significantly enhance prediction accuracy. In the data preprocessing stage, essential feature variables were selected based on their relevance to T2DM. Following feature selection, irrelevant features were removed through data dimensionality reduction to improve the prediction model’s accuracy and reduce runtime. Finally, a total of 28 candidate variables were selected to identify a 30-day readmission, and these included 7 demographic variables, 8 comorbidities, 3 drug types, and 10 laboratory parameters. Specifically, we explored age, sex, past surgical history (PSH), smoking history, drinking history, emergency department visits (ESVs), length of stay (LOS), age-adjusted charlson comorbidity index (ACCI) score, hypertension, coronary heart disease (CHD), cerebral infarction (CI), hyperlipidemia, chronic gastritis, osteoporosis, pulmonary infections, total cholesterol (TC), triglycerides (TGs), creatinine (CREA), uric acid (UA), low density lipoprotein cholesterol (LDL-C), high density lipoprotein cholesterol (HDL-C), glycated hemoglobin (HBA1c), fasting glucose (FG), estimated glomerular filtration rate (eGFR), neutrophil percentage-to-albumin ratio (NPAR), anti-hypertensive drug use, statin use, and antiplatelet and anticoagulant use. Medication subtypes were aggregated into broader categories (e.g., antihypertensive drugs) to ensure the model’s generalizability and avoid overfitting, given the limited sample size. These decisions were guided by statistical considerations and clinical relevance, ensuring that the final model is both robust and applicable to clinical practice. **Supplementary Table S1** presents the variance inflation factors (VIFs) for the 28 selected variables. All variables have VIFs less than 5, indicating no significant multicollinearity.

### 2.5 Statistical analyses

Enumerative data were expressed as frequencies and percentages, and the chi-square test was used for group comparisons. Quantitative variables, which did not follow a normal distribution, were represented by median and interquartile range (IQR), and the nonparametric rank-sum test was used for group comparisons. To maximize statistical power and minimize bias that might occur if patient with missing data were excluded from analyses, we used multivariate multiple imputation with chained equations to impute missing values.

First, univariate analyses were conducted to evaluate the association between each of the 28 initial predictors and the outcome. Variables with a p-value < 0.05 in the univariate analysis were retained for further consideration. Second, multicollinearity among the retained variables was assessed using VIF, with a threshold of VIF less than 5 indicating no significant multicollinearity. The correlation matrix was utilized to further illustrate the relationships between variables. Third, least absolute shrinkage and selection operator (LASSO) regression and multivariable logistic regression analyses were employed to identify the independent predictors (22, 23). The model was assessed using holdout data with the area under the receiver operating characteristic curve (AUROC), calibration curves, decision curve analysis (DCA), and clinical impact curves (CIC) (24, 25). The DCA is a type of chart that effectively illustrates net benefits across a range of reasonable risk thresholds. The CIC was developed based on the DCA to visually display the estimated number of high-risk patients associated with each risk threshold. Additionally, XGBoost, LightGBM, Decision Tree (DT), and Random Forest (RF) algorithms were employed to assess the predictive performance of the logistic regression (LR) model. A difference with *P* < 0.05 was considered statistically significant. Statistical analyses were performed using SPSS 22.0 and R (version 4.0.2, Vienna, Austria).

## 3 Results

### 3.1 Patient characteristics

A total of 1,899 patients were included in the study, with 955, 409, and 535 in the derivation, internal validation, and external validation cohorts, respectively. In the derivation and internal validation cohorts, the median age was 76.00 years old (IQR: 71.00-81.00), and 56.01% were female. 30.87% of patients had a smoking history, 23.39% had a drinking history, and 84.60% had hypertension. Within 30 days, 169 patients (12.39%) were readmitted (**Table 1**). Furthermore, the Mann-Whitney U test indicated no significant differences in the missing variables between the derivation and internal validation cohorts, both before and after multiple imputations (**Supplementary Table S2**). **Supplementary Table S3** displays data from the five hospitals. In addition, the 30-day readmission rates varied significantly among different classes of anti-hypertensive medications (**Supplementary Table S4**). Patients taking diuretics had the highest readmission rate (19.12%), followed by those taking β blockers (13.04%) and angiotensin receptor blockers (12.61%).

**Table 1:** Baseline characteristics of elderly T2DM patients complicated with HF.

Variables	Derivation cohort (N=955)	Internal validation cohort (N=409)	Total (N=1,364)
Demographics			
age (IQR, year)	76.00 (70.50,81.00)	76.00 (71.00,82.00)	76.00 (71.00,81.00)
sex (n, %)			
female	518 (54.24)	246 (60.15)	764 (56.01)
male	437 (45.76)	163 (39.85)	600 (43.99)
PSH (n, %)			
no	332 (34.76)	134 (32.76)	466 (34.16)
yes	623 (65.24)	275 (67.24)	898 (65.84)
smoking history (n, %)			
no	646 (67.64)	297 (72.62)	943 (69.13)
yes	309 (32.36)	112 (27.38)	421 (30.87)
drinking history (n, %)			
no	721 (75.50)	324 (79.22)	1,045 (76.61)
yes	234 (24.50)	85 (20.78)	319 (23.39)
EDVs (n, %)			
0	574 (60.10)	257 (62.84)	831 (60.92)
≥1	381 (39.90)	152 (37.16)	533 (39.08)
LOS (n, %)			
2-9	442 (46.28)	196 (47.92)	638 (46.77)
≥10	513 (53.72)	213 (52.08)	726 (53.23)
Comorbidities			
ACCI score (n, %)			
0-6	320 (33.51)	142 (34.72)	462 (33.87)
≥7	635 (66.49)	267 (65.28)	902 (66.13)
hypertension (n, %)			
no	156 (16.34)	54 (13.20)	210 (15.40)
yes	799 (83.66)	355 (86.80)	1,154 (84.60)
CHD (n, %)			
no	281 (29.42)	117 (28.61)	398 (29.18)
yes	674 (70.58)	292 (71.39)	966 (70.82)
CI (n, %)			
no	729 (76.34)	318 (77.75)	1,047 (76.76)
yes	226 (23.66)	91 (22.25)	317 (23.24)
hyperlipidemia (n, %)			
no	778 (81.47)	310 (75.79)	1,088 (79.77)
yes	177 (18.53)	99 (24.21)	276 (20.23)
chronic gastritis (n, %)			
no	848 (88.80)	352 (86.06)	1,200 (87.98)
yes	107 (11.20)	57 (13.94)	164 (12.02)
osteoporosis (n, %)			
no	819 (85.76)	350 (85.57)	1,169 (85.70)
yes	136 (14.24)	59 (14.43)	195 (14.30)
pulmonary infections (n, %)			
no	841 (88.06)	374 (91.44)	1,215 (89.08)
yes	114 (11.94)	35 (8.56)	149 (10.92)
Drug types			
antihypertensive drug use (n, %)			
No	93 (9.74)	37 (9.05)	130 (9.53)
Yes	862 (90.26)	372 (90.95)	1,234 (90.47)
statin use (n, %)			
No	281 (29.42)	124 (30.32)	405 (29.69)
Yes	674 (70.58)	285 (69.68)	959 (70.31)
antiplatelet and anticoagulant use (n, %)			
No	176 (18.43)	64 (15.65)	240 (17.60)
Yes	779 (81.57)	345 (84.35)	1,124 (82.40)
Laboratory parameters			
TC (IQR, mmol/l)	4.25 (3.49,5.03)	4.28 (3.60,5.07)	4.26 (3.52,5.04)
TGs (IQR, mmol/l)	1.37 (0.99,2.02)	1.43 (1.02,1.94)	1.39 (1.00,1.99)
CREA (IQR, umol/l)	79.30 (64.20,101.45)	76.30 (60.10,98.50)	78.60 (62.97,100.20)
UA (IQR, umol/l)	348.20 (285.25,426.46)	352.00 (282.90,411.90)	350.20 (284.60,422.03)
LDL-C (IQR, mmol/l)	2.34 (1.78,3.03)	2.37 (1.80,3.05)	2.35 (1.79,3.04)
HDL-C (IQR, mmol/l)	1.07 (0.91,1.27)	1.09 (0.93,1.33)	1.08 (0.91,1.29)
HbA1c (IQR, %)	7.20 (6.40,8.30)	7.30 (6.50,8.60)	7.20 (6.50,8.40)
FG (IQR, mmol/l)	7.66 (5.97,10.72)	7.65 (6.01,10.40)	7.66 (5.97,10.63)
eGFR (IQR, ml/min)	75.98 (53.69,93.43)	77.03 (57.07,98.51)	76.53 (55.02,94.31)
NPAR (IQR, ml/g)	17.69 (15.37,20.36)	17.25 (15.29,19.77)	17.59 (15.35,20.20)

PSH, past surgical history; EDVs, emergency department visits; LOS, length of stay; ACCI, age-adjusted Charlson comorbidity index; CHD, coronary heart disease; CI, cerebral infarction; TC, total cholesterol; TGs, triglycerides; CREA, creatinine; UA, uric acid; LDL-C, low density lipoprotein cholesterol; HDL-C, high density lipoprotein cholesterol; HbA1c, glycated hemoglobin; FG, fasting glucose; eGFR, estimated glomerular filtration rate; NPAR, neutrophil percentage-to-albumin ratio; IQR, interquartile range.

### 3.2 Selection of predictors

Patients in the derivation cohort were divided into readmission and non-readmission groups. The following factors were significantly associated with 30-day readmission in univariate analysis: age (*P* = 0.039), PSH (*P* = 0.021), osteoporosis (*P* = 0.049), pulmonary infections (*P* < 0.001), anti-hypertensive drug use (*P* = 0.005), CREA (*P* = 0.002), eGFR (*P* = 0.001), and NPAR (*P* < 0.001) (**Table 2**). **Supplementary Figures S2** illustrated the distribution and relationships of individual variables with 30-day readmission. VIFs and correlation matrix also showed low level of co-linearity for all independent variables (**Supplementary Table S5**, **Supplementary Figures S3**).

**Table 2:** Demographic and clinical characteristics associated with readmission as assessed in the derivation cohort.

Variables	Total (N=955)	Readmission (N=124)	Non-readmission (N=831)	*P* values
Demographics				
age (IQR, year)	76.00 (70.50,81.00)	77.00 (72.00,82.00)	76.00 (70.00,81.00)	0.039
sex (n, %)				0.734
female	518 (54.24)	65 (52.42)	453 (54.51)	
male	437 (45.76)	59 (47.58)	378 (45.49)	
PSH (n, %)				0.021
no	332 (34.76)	55 (44.35)	277 (33.33)	
yes	623 (65.24)	69 (55.65)	554 (66.67)	
smoking history (n, %)				0.777
no	646 (67.64)	82 (66.13)	564 (67.87)	
yes	309 (32.36)	42 (33.87)	267 (32.13)	
drinking history (n, %)				1.000
no	721 (75.50)	94 (75.81)	627 (75.45)	
yes	234 (24.50)	30 (24.19)	204 (24.55)	
EDVs (n, %)				1.000
0	574 (60.10)	75 (60.48)	449 (60.05)	
≥1	381 (39.90)	49 (39.52)	332 (39.95)	
LOS (n, %)				0.183
2~9	442 (46.28)	50 (40.32)	392 (47.17)	
≥10	513 (53.72)	74 (59.68)	439 (52.83)	
Comorbidities				
ACCI score (n, %)				0.217
0~6	320 (33.51)	35 (28.23)	285 (34.30)	
≥7	635 (66.49)	89 (71.77)	546 (65.70)	
hypertension (n, %)				0.269
no	156 (16.34)	25 (20.16)	131 (15.76)	
yes	799 (83.66)	99 (79.84)	700 (84.24)	
CHD (n, %)				0.528
no	281 (29.42)	33 (26.61)	248 (29.84)	
yes	674 (70.58)	91 (73.39)	583 (70.16)	
CI (n, %)				1.000
no	729 (76.34)	95 (76.61)	634 (76.29)	
yes	226 (23.66)	29 (23.39)	197 (23.71)	
hyperlipidemia (n, %)				0.108
no	778 (81.47)	108 (87.10)	670 (80.63)	
yes	177 (18.53)	16 (12.90)	161 (19.37)	
chronic gastritis (n, %)				0.624
no	848 (88.80)	108 (87.10)	740 (89.05)	
yes	107 (11.20)	16 (12.90)	91 (10.95)	
osteoporosis (n, %)				0.049
no	819 (85.76)	114 (91.94)	705 (84.84)	
yes	136 (14.24)	10 (8.06)	126 (15.16)	
pulmonary infections (n, %)				<0.001
no	841 (88.06)	81 (65.32)	760 (91.46)	
yes	114 (11.94)	43 (34.68)	71 (8.54)	
Drug types				
anti-hypertensive drug use (n, %)				0.005
no	93 (9.74)	3 (2.42)	90 (10.83)	
yes	862 (90.26)	121 (97.58)	741 (89.17)	
statin use (n, %)				0.524
no	281 (29.42)	40 (32.26)	241 (29.00)	
yes	674 (70.58)	84 (67.74)	590 (71.00)	
antiplatelet and anticoagulant use (n, %)				1.000
no	176 (18.43)	23 (18.55)	153 (18.41)	
yes	779 (81.57)	101 (81.45)	678 (81.59)	
Laboratory parameters				
TC (IQR, mmol/l)	4.25 (3.49,5.03)	4.20 (3.29,4.96)	4.26 (3.52,5.04)	0.353
TGs (IQR, mmol/l)	1.37 (0.99,2.02)	1.32 (0.95,1.94)	1.38 (1.01,2.02)	0.257
CREA (IQR, umol/l)	79.30 (64.20,101.45)	86.25 (69.01,122.70)	78.10 (63.75,99.20)	0.002
UA (IQR, umol/l)	348.20 (285.25,426.46)	361.90 (303.91,449.94)	346.50 (284.30,422.22)	0.087
LDL-C (IQR, mmol/l)	2.34 (1.78,3.03)	2.25 (1.75,2.87)	2.36 (1.79,3.06)	0.391
HDL-C (IQR, mmol/l)	1.07 (0.91,1.27)	1.06 (0.86,1.30)	1.08 (0.91,1.27)	0.764
HbA1c (IQR, %)	7.20 (6.40,8.30)	7.10 (6.49,8.60)	7.20 (6.40,8.30)	0.924
FG (IQR, mmol/l)	7.66 (5.97,10.72)	7.50 (6.03,11.04)	7.66 (5.95,10.62)	0.653
eGFR (IQR, ml/min)	75.98 (53.69,93.43)	71.32 (40.55,85.91)	76.71 (56.34,94.13)	0.001
NPAR (IQR, ml/g)	17.69 (15.37,20.36)	21.09 (18.14,24.33)	17.42 (15.27,19.71)	<0.001

PSH, past surgical history; EDVs, emergency department visits; LOS, length of stay; ACCI, age-adjusted Charlson comorbidity index; CHD, coronary heart disease; CI, cerebral infarction; TC, total cholesterol; TGs, triglycerides; CREA, creatinine; UA, uric acid; LDL-C, low density lipoprotein cholesterol; HDL-C, high density lipoprotein cholesterol; HbA1c, glycated hemoglobin; FG, fasting glucose; eGFR, estimated glomerular filtration rate; NPAR, neutrophil percentage-to-albumin ratio; IQR, interquartile range.

**Figure 1:**
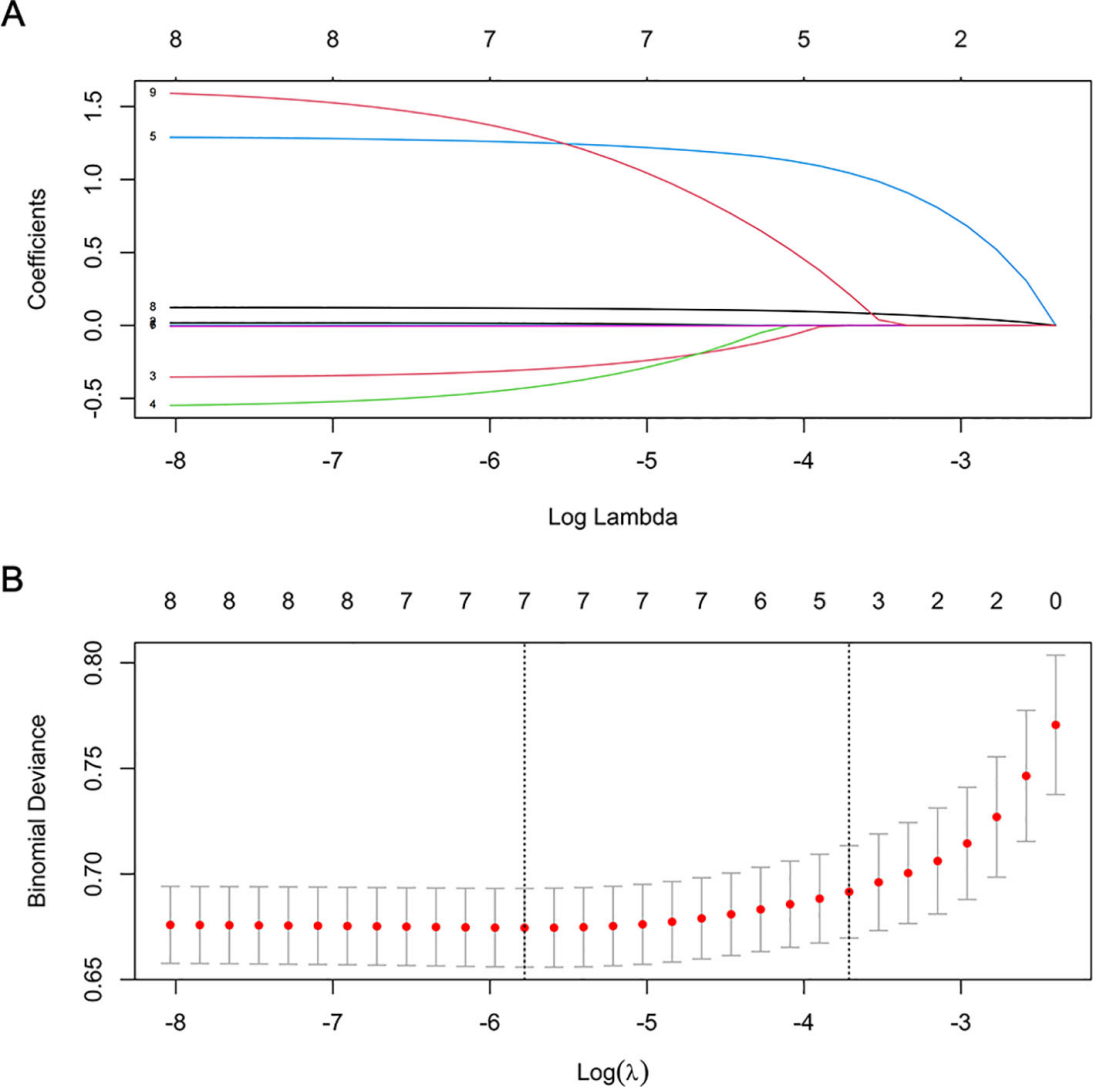
Features selection by LASSO. **(A)** LASSO coefficient profiles (y-axis) of 8 features. The upper x-axis is the average numbers of predictors and the lower x-axis is the log(λ). **(B)** Tenfold cross-validation for tuning parameter selection in the LASSO model.

**Figure 2:**
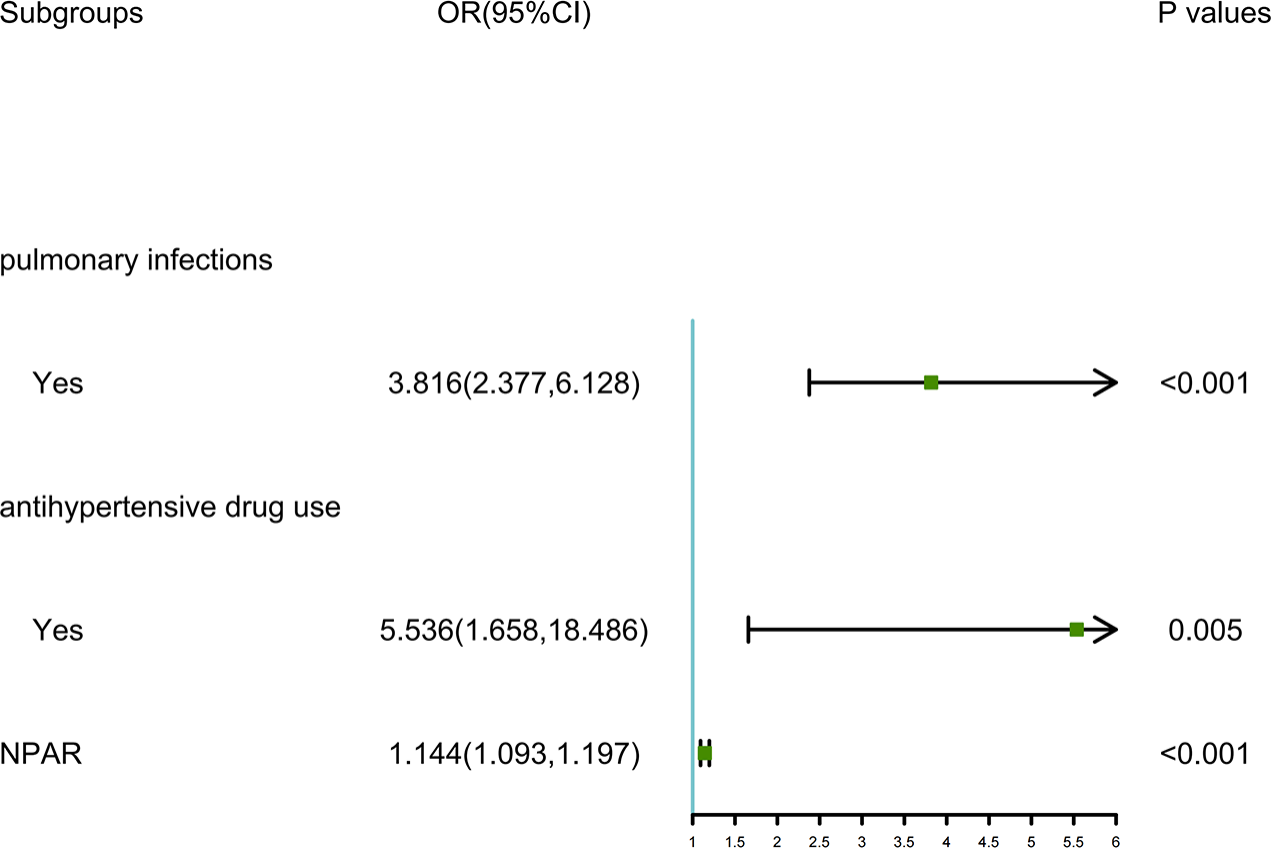
Forest plot showing the results of multivariable analysis.

As depicted in **Figure 1**, the LASSO regression selected the optimal lambda value of 0.02443081, which minimized the cross-validation error and identified three key predictors: pulmonary infections, anti-hypertensive drug use, and NPAR. These variables were chosen because they exhibited the strongest associations with 30-day readmission risk while maintaining model parsimony. Ultimately, the multivariate logistic regression model further indicated that pulmonary infections (odds ratio [OR]: 3.816, 95% confidence interval [CI]: 2.377-6.128, *P* < 0.001), anti-hypertensive drug use (OR: 5.536, 95% CI: 1.658-18.486, *P* = 0.005), and NPAR (OR: 1.144, 95% CI: 1.093-1.197, *P* < 0.001) were independent predictors of 30-day readmission risk (**Figure 2**). We also evaluated variable subsets with the top K (ranging from 1 to 8) features to further validate the performance of LASSO-logistic regression in screening predictors. Finally, after identified three variables with the highest information gain, adding additional variables did not result in a significant increase in the AUROC (rolling mean *P* = 0.671, **Supplementary Figure S4**), which was consistent with the LASSO-logistic regression model. This finding also indicated that adding more variables, even those closely related to 30-day readmission, may not necessarily improve the predictive performance of the model (**Supplementary Table S6**).

### 3.3 Model comparison

The best-performing model referring to the derivation cohort was XGBoost, with an AUROC of 0.912; the sensitivity and specificity were 0.887 and 0.794, respectively. The best-performing model referring to the internal validation cohort was DT, with an AUROC of 0.762; the sensitivity and specificity were 0.644 and 0.879, respectively. The best-performing model referring to the external validation cohort was LR, with an AUROC of 0.753; the sensitivity and specificity were 0.646 and 0.825, respectively. The AUROC, sensitivity, and specificity of each model in the derivation, internal, and external validation cohorts were comprehensively analyzed in **Table 3**. Considering the performance, complexity, generalization ability, and practicality, the LR model demonstrates better stability, while the XGBoost and LightGBM models appear to exhibit overfitting.

**Table 3:** Performance of machine learning models.

Cohorts	Models	AUROC (95%CI)	Sensitivity (95%CI)	Specificity (95%CI)
derivation cohort				
	RF	0.678 (0.628-0.727)	0.718 (0.639-0.797)	0.629 (0.597-0.662)
	DT	0.752 (0.707-0.796)	0.677 (0.595-0.760)	0.809 (0.782-0.835)
	XGBoost	0.912 (0.886-0.937)	0.887 (0.831-0.943)	0.794 (0.767-0.822)
	lightGBM	0.865 (0.835-0.896)	0.798 (0.728-0.869)	0.787 (0.759-0.815)
	LR	0.782 (0.737-0.826)	0.669 (0.587-0.752)	0.824 (0.798-0.850)
internal validation cohort				
	RF	0.707 (0.629-0.784)	0.711 (0.579-0.844)	0.665 (0.616-0.713)
	DT	0.762 (0.689-0.835)	0.644 (0.505-0.784)	0.879 (0.846-0.913)
	XGBoost	0.708 (0.615-0.800)	0.689 (0.554-0.824)	0.731 (0.685-0.776)
	lightGBM	0.719 (0.643-0.796)	0.533 (0.388-0.679)	0.849 (0.812-0.886)
	LR	0.746 (0.654-0.838)	0.644 (0.505-0.784)	0.874 (0.839-0.908)
external validation cohort				
	RF	0.622 (0.552-0.693)	0.392 (0.285-0.500)	0.853 (0.821-0.886)
	DT	0.636 (0.578-0.694)	0.430 (0.321-0.540)	0.838 (0.804-0.872)
	XGBoost	0.707 (0.641-0.774)	0.557 (0.447-0.667)	0.774 (0.736-0.813)
	lightGBM	0.670 (0.600-0.740)	0.506 (0.396-0.617)	0.798 (0.761-0.835)
	LR	0.753 (0.684-0.813)	0.646 (0.540-0.751)	0.825 (0.790-0.859)

RF, random forest; DT, decision tree; LR, logistic regression; AUROC, area under the receiver operating characteristic curve; CI, Confidence Interval.

### 3.4 Nomogram construction and performance

**Figure 3:**
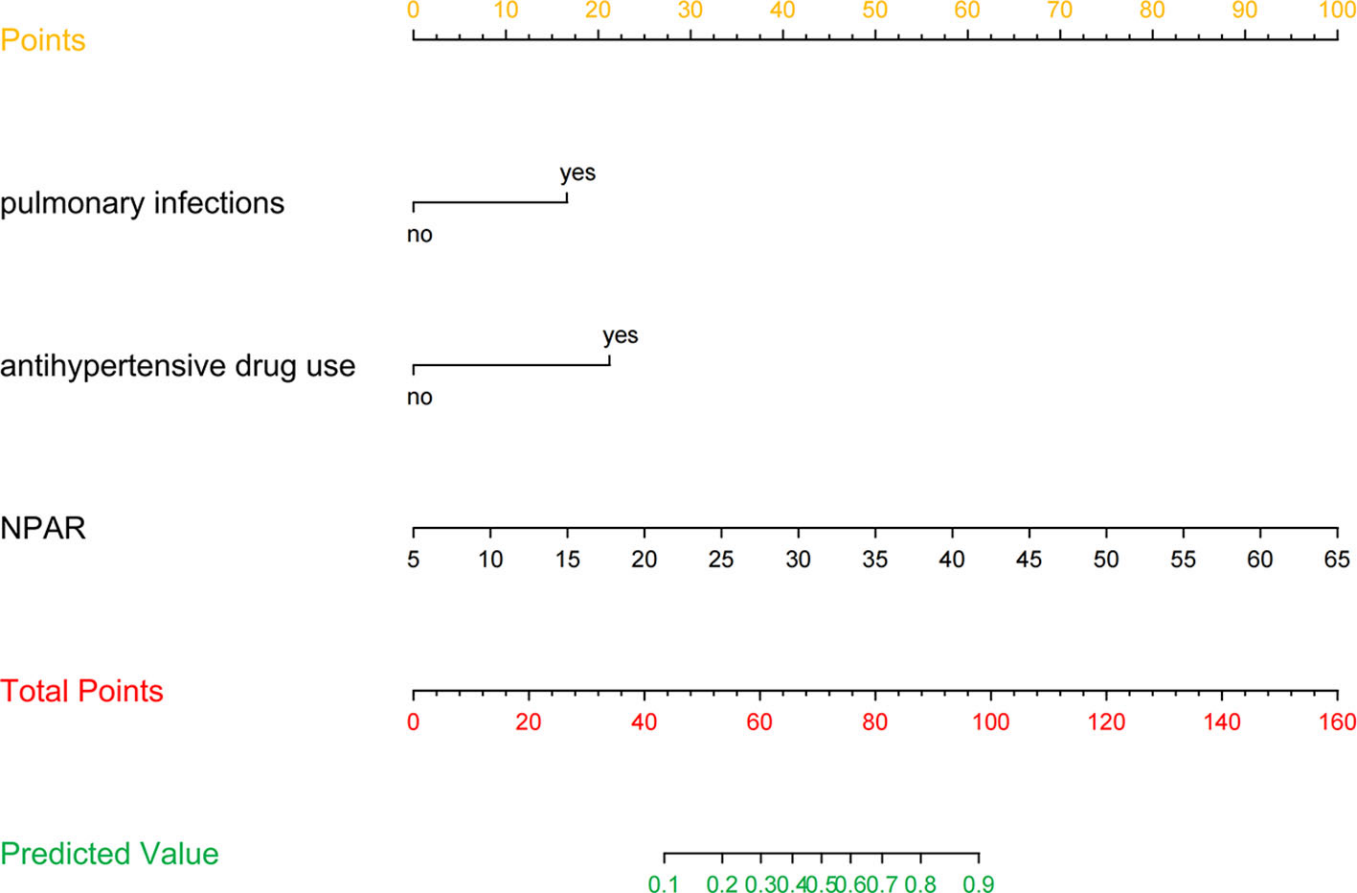
Nomogram predicting 30-day readmission in elderly T2DM patients complicated with HF. The values of each variable were scored between 0 and 100, then added to give the total points score which was then used to predict the probability of 30-day readmission.

**Figure 4:**
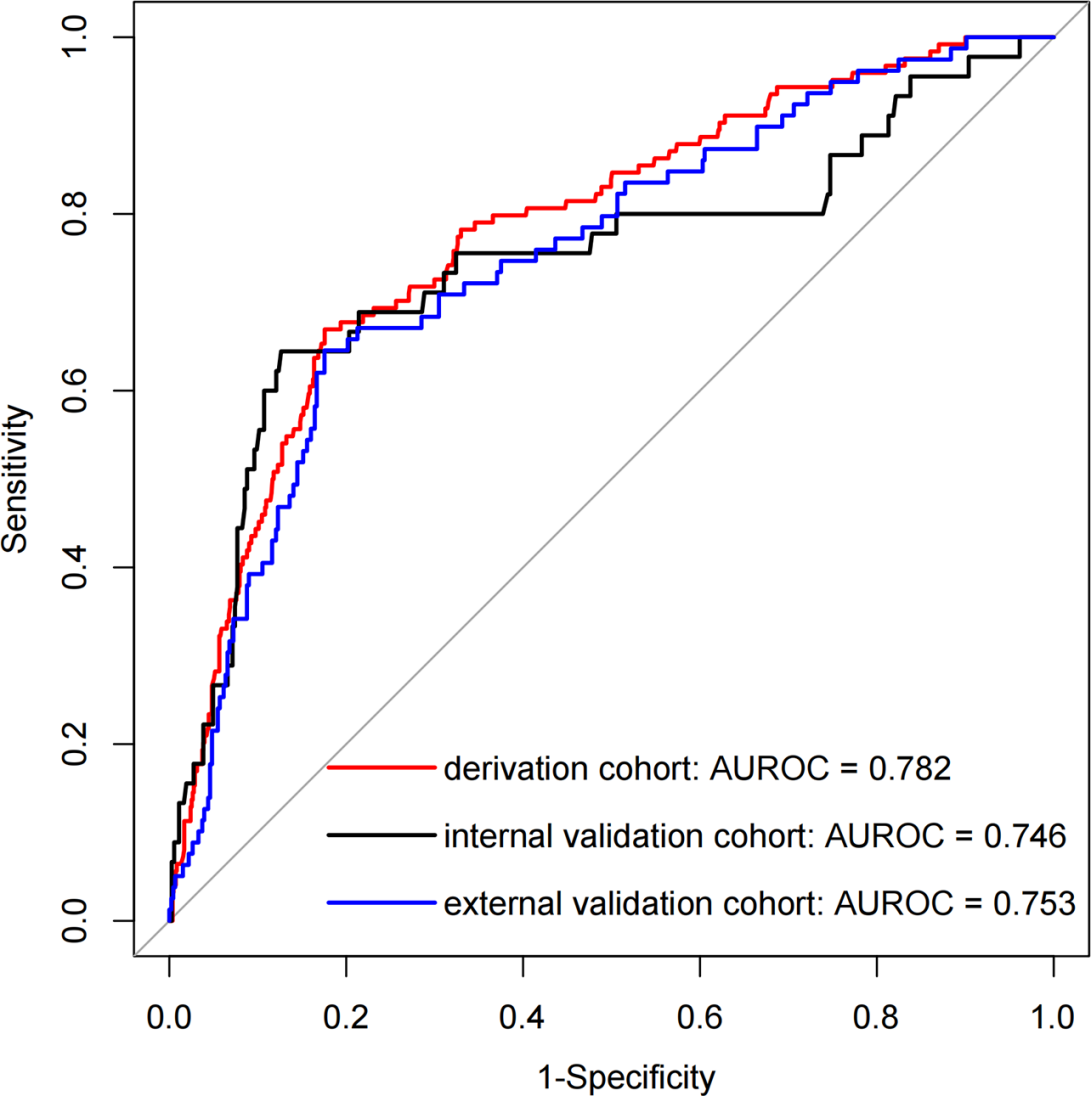
The AUROC curves of the nomogram for predicting 30-day readmission in elderly T2DM patients complicated with HF.

**Figure 5:**
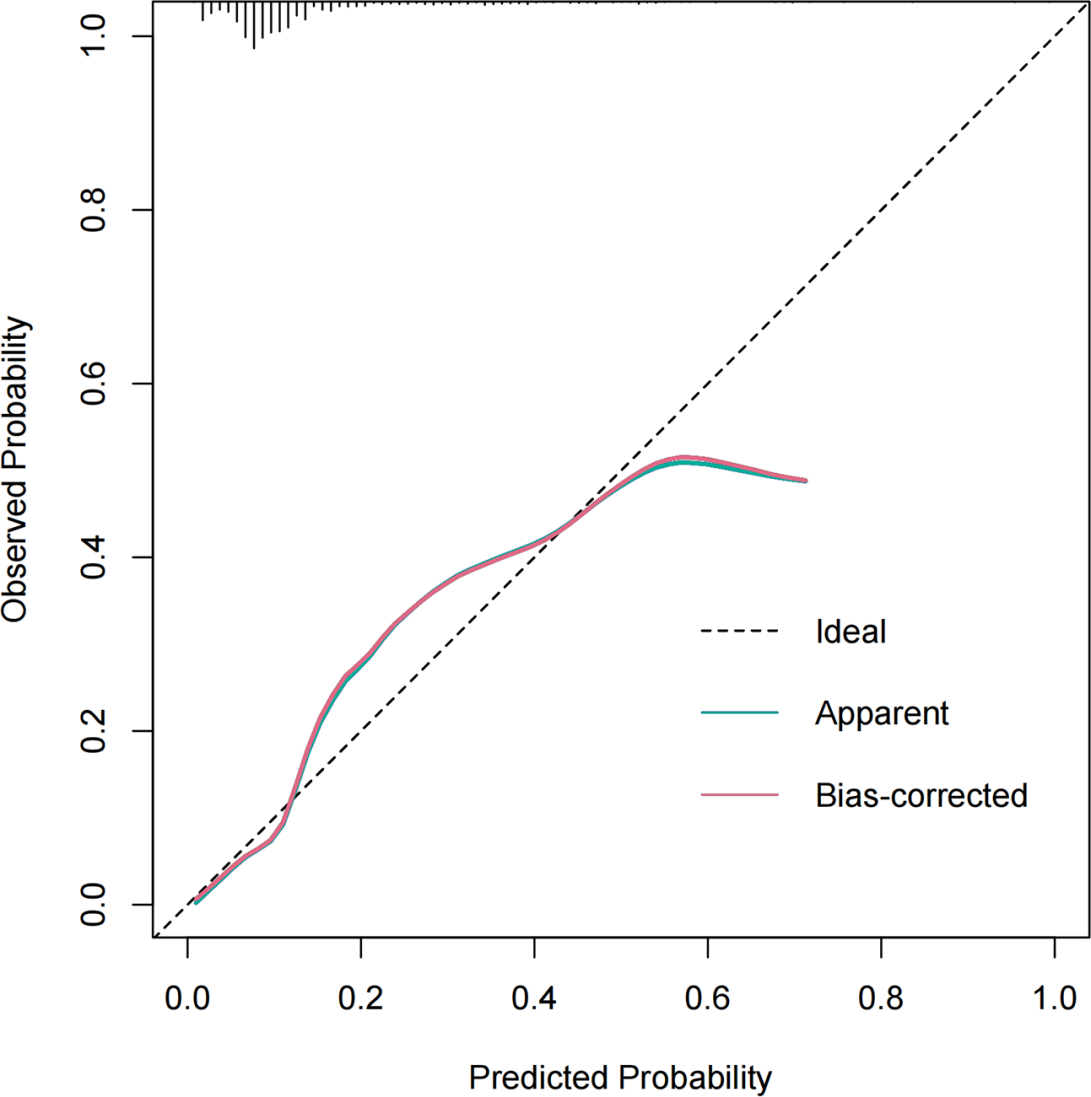
Calibration curve of the model in the derivation cohort. The x-axis represents the predicted probability of 30-day readmission. The y-axis represents the actual occurred 30-day readmission. the black dashed line represents the perfect prediction with the same predicted probability as the actual probability. The blue dashed line represents the performance of the nomogram and the red solid line represents the performance of the model after calibration. The closer the calibration curve of the model is to the black dashed line, the better the model prediction is represented.

**Figure 3** presents the LR model as a nomogram for calculating the probability of 30-day readmission in elderly T2DM patients complicated with HF. The model displayed a high predictive ability, with an AUROC of 0.782 (95% CI: 0.737-0.826) in the derivation cohort, 0.746 (95% CI: 0.654-0.838) in the internal validation cohort, and 0.753 (95% CI: 0.684-0.813) in the external validation cohort (**Figure 4**). The best cutoff value was 0.138. The calibration curve (bootstraps = 1,000) indicated good calibration (**Figure 5**). **Supplementary Figures S5**-**S6** display the calibration curves for the internal and external validation cohorts, respectively.

### 3.5 Clinical utility of the nomogram

**Figure 6:**
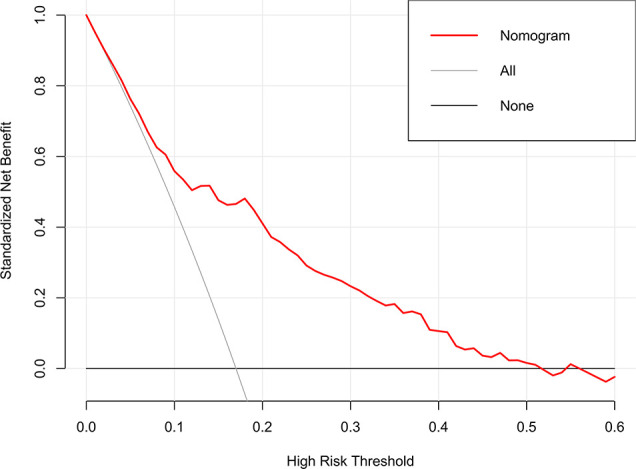
DCA of the model in the derivation cohort. The net benefits were measured at different threshold probabilities. The gray line represents the assumption that all patients are identified as 30-day readmission. The black line represents the assumption that no patients are identified as 30-day readmission.

**Figure 7:**
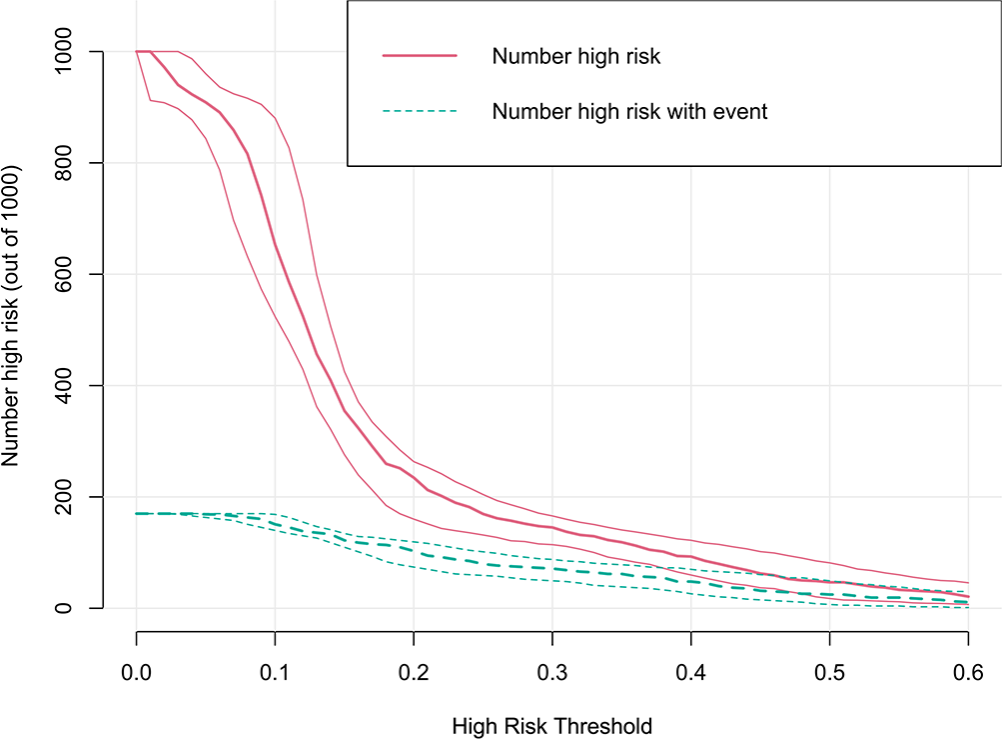
CIC of the model in the derivation cohort. The clinical impact curve illustrated the number of 30-day readmission in a sample population of 1000. The blue curve indicates the predicted number of 30-day readmission at various threshold probabilities, while the red curve represents the actual number of 30-day readmission.

The clinical utility of the model was evaluated by DCA, indicating that the model has superior clinical application value in predicting 30-day readmission of elderly T2DM patients with HF (**Figure 6**). Meanwhile, the CIC intuitively indicated that the model has superior overall net returns within the threshold probability range (**Figure 7**). **Supplementary Figures S7**-**S8** display the DCAs for the internal and external validation cohorts, respectively. **Supplementary Figures S9**-**S10** display the CICs for the internal and external validation cohorts, respectively.

### 3.6 Construction of web app to easily access the nomogram

**Figure 8:**
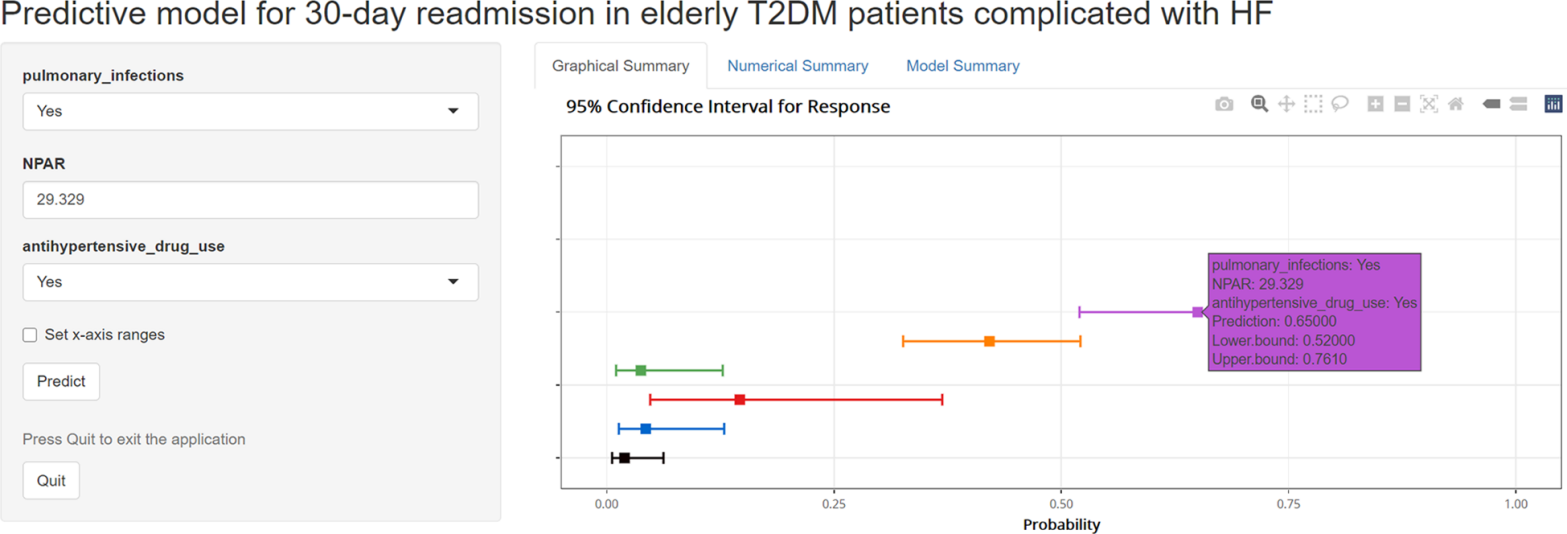
An example of model to predicting 30-day readmission in elderly T2DM patients complicated with HF via a link.

To facilitate the use of the model by clinicians, we created an online interface (https://cqykdxtjt.shinyapps.io/readmission/) to calculate the exact probability of 30-day readmission. For example, if a patient has pulmonary infections, anti-hypertensive drug use, and NPAR level of 29.329 ml/g, the probability of 30-day readmission would be 0.650 (95% CI: 0.520-0.761) (**Figure 8**). To ensure the tool remains accurate and relevant, we have established a plan for ongoing model monitoring and updating. This includes regular performance evaluations using new data, with updates to the model parameters as needed. The monitoring process will involve tracking key performance metrics (e.g., AUROC, calibration) and comparing them to predefined thresholds. Moreover, to address potential population drift, we have implemented a strategy for detecting and adjusting to changes in the patient population over time. This includes regular monitoring of demographic and clinical characteristics to identify shifts in the population.

## 4 Discussion

In this study, we assessed several candidate variables potentially associated with an increased risk of 30-day readmission in elderly T2DM patients complicated with HF. Our findings demonstrated a straightforward predictive model based on three predictors (pulmonary infections, anti-hypertensive drug use, and NPAR) could effectively identify underlying 30-day readmission, with an AUROC of 0.782 (95% CI: 0.737-0.826).

Zhang et al. used machine learning (ML) to predict the 30-day readmission risk of patients with acute HF, with a distinguishability of 0.763 (26). A study conducted in Canada compared ML models with conventional statistical methods to predict 30-day readmission for both cardiovascular and non-cardiovascular causes in HF patients. The results demonstrated that the discrimination was modest in all readmission prediction models regardless of the methods used (27). Hu et al. used ML algorithms to predict the mortality and 30-day readmission in ICU patients with T2DM. The results indicated that MLP and AdaBoost models had the highest performance in predicting the 30-day readmission, with an AUROC value of 0.849 and an accuracy of 0.925 (28). Most studies focus on single diseases (e.g., HF or diabetes) rather than comorbid conditions, such as T2DM complicated with HF, which is a key focus of our study.

This study found that pulmonary infection was an independent risk factor for 30-day readmission in elderly T2DM patients complicated with HF, consistent with previous studies (29, 30). T2DM patients complicated with HF experience pulmonary congestion and edema, leading to breathing difficulties and impaired gas exchange, which create favorable conditions for pathogen invasion and colonization in the lungs (31, 32). When the lungs of T2DM patients complicated with HF are infected, the cardiac load increases while the patient’s respiratory ventilation decreases, resulting in reduced myocardial oxygen supply and further exacerbating the cardiac load, worsening the patient’s condition and leading to readmission. In a multicenter prospective study involving 8,099 HF patients, 21% of patients were vaccinated against respiratory influenza, and the results showed that vaccination reduced the probability of pulmonary infections, thereby decreasing mortality and readmission rates (33). Researches suggested that the three most common pathogens of pulmonary infections were Pseudomonas aeruginosa, *Staphylococcus aureus*, and Klebsiella pneumoniae (34, 35). Therefore, during the treatment period, strict monitoring of sputum and bacterial cultivation should be conducted. Medical staff should guide patients to consume high-calorie, high-protein, and high-vitamin foods, advise them to elevate the bedside and rest in bed properly, and encourage family members to assist in daily activities (36, 37). Actions such as back tapping, phlegm expulsion, and repositioning are beneficial for preventing pulmonary infections (38). In summary, the strong correlation between pulmonary infection and 30-day readmission underscores the necessity for proactive measures to prevent respiratory infections in elderly T2DM patients complicated with HF. Clinicians should consider implementing vaccination programs (e.g., influenza and pneumococcal vaccines) and educating patients on respiratory hygiene practices. Furthermore, early detection and aggressive management of pulmonary infections during hospitalization may reduce the risk of 30-day readmission.

NPAR is a novel indicator of systemic inflammation and malnutrition. Previous studies have demonstrated that NPAR can serve as a prognostic marker for diseases such as acute myocardial infarction, sepsis or septic shock, acute kidney injury, and T2DM (39–41). Several mechanisms may underlie the association between high NPAR and poor prognosis in patients with HF. Firstly, neutrophil play a crucial role in the pathogenesis and progression of HF. They mediate thrombus formation in myocardial blood vessels through the KLF2/NETosis pathway, leading to myocardial hypoxia, cell death, and hypertrophy. This neutrophil-mediated immune thrombosis is a key mechanism of HF (42). Meanwhile, neutrophil is also an important marker of vascular endothelial dysfunction, which can further exacerbate circulatory pressure in HF patients (43). Secondly, serum albumin possesses antioxidant and anti-inflammatory properties, as well as the ability to bind various molecules and drugs (44). In HF patients, a low albumin level can indicate malnutrition, decreased liver synthesis, increased venous pressure, and visceral congestion (45–47). Kato et al. analyzed follow-up data from 3,160 hospitalized HF patients and found that those with elevated serum albumin levels (n=1,083, 34%) had a lower risk of death and readmission (HR=0.78, P < 0.001) compared to patients without increased serum albumin levels (n=2,077, 66%) (48). Another cohort study investigated 5,779 HF patients, of which 12% had hypoproteinemia (< 35g/L). Cox regression analysis showed that lower serum albumin level led to higher mortality rates, and the study also highlighted that serum albumin level was significant predictor of patient readmission (49). Therefore, it is recommended to include NPAR in routine admission testing for hospitalized T2DM patients complicated with HF. Hence, this finding implies that targeting inflammation and thrombosis may be an effective strategy to reduce 30-day readmission rates. For instance, anti-inflammatory agents or anticoagulants might be considered for patients with elevated NPAR levels, though further research is necessary to assess the efficacy and safety of these interventions.

Interestingly, our findings revealed that anti-hypertensive drug use (diuretics, calcium channel blockers, β blockers, and others) was associated with 30-day readmission in elderly T2DM patients complicated with HF. Specifically, patients using diuretics exhibited the highest 30-day readmission rate (48/251, 19.12%), followed by those using β-blockers (21/161, 13.04%). These differences may be attributed to the varying effects of these medications on heart failure management, fluid balance, and renal function. For example, ACE inhibitors are known to improve outcomes in heart failure patients by reducing afterload and preventing ventricular remodeling, whereas diuretics, while effective in managing fluid overload, may lead to electrolyte imbalances and renal dysfunction, potentially increasing the risk of readmission (50, 51). However, the relationship between anti-hypertensive drugs and 30-day readmission is complex, as both over-treatment and under-treatment can result in adverse outcomes. Future research should investigate the optimal blood pressure targets and anti-hypertensive regimens for this population. In clinical practice, regular monitoring and individualized adjustment of anti-hypertensive therapy may help reduce 30-day readmission rates.

In comparison to previous studies, this study enhances the evaluation tools for 30-day readmission rates in elderly T2DM patients complicated with HF, providing valuable references for clinical and nursing care. The study has two primary advantages: first, it utilizes a large sample size and multicenter data to construct the prediction model; second, the variables employed in the model are simple and easily obtainable, enhancing the model’s generalizability and facilitating its clinical application. However, there are some limitations. First, the retrospective design may introduce selection bias, as the data were collected from existing records rather than through a controlled experimental setup. Second, we only analyzed the 30-day readmission of elderly T2DM patients with HF and recommend extending the follow-up period in future studies to further explore the long-term prognosis of these patients. Third, the potential for missing data represents a significant limitation, as it could lead to biased estimates if the data are not missing at random. To address this, we employed multivariate multiple imputation to handle missing data and conducted complete-case analyses to compare the results. Nonetheless, the possibility of residual bias cannot be entirely ruled out. Fourth, the inclusion and exclusion criteria may have resulted in a study population that is not fully representative of the broader patient population. For example, patients with severe comorbidities or those lost to follow-up may have been excluded, potentially biasing the results. The limitations of our study may affect its clinical applicability. Future prospective studies with standardized data collection protocols are required to validate our findings and provide stronger evidence for clinical practice.

## 5 Conclusion

This study analyzed the factors influencing 30-day readmission in elderly T2DM patients complicated with HF using EMR data and developed a predictive model to assess readmission risk. Through LASSO regression and multivariate logistic regression analysis, pulmonary infections, anti-hypertensive drug use, and NPAR were identified as independent predictors of 30-day readmission. The predictive model demonstrated good discriminative ability, with an AUROC of 0.782 in the derivation cohort, 0.746 in the internal validation cohort, 0.753 in the external validation cohort, highlighting its potential for clinical application. Our findings underscore the importance of early evaluation and risk prediction before discharge, particularly for elderly patients with multiple comorbidities. Clinicians should prioritize the management of pulmonary infections, optimize anti-hypertensive therapy, and monitor NPAR levels to reduce readmission risk. Additionally, discharge health education should include personalized guidance on medication adherence, lifestyle modifications, and symptom monitoring to empower patients and caregivers.

## Data Availability

The raw data supporting the conclusions of this article will be made available by the authors, without undue reservation.
