# Supplementary material for: Development and validation of a risk prediction model for 30-day readmission in elderly type 2 diabetes patients complicated with heart failure: a multicenter, retrospective study
Source: Front Endocrinol (Lausanne). 2025 Feb 27;16:1534516. doi: 10.3389/fendo.2025.1534516 (PMC11903290; doi:10.3389/fendo.2025.1534516)
Supplement: Supplementary file 1 [file DataSheet1.docx]

**Supplementary information**

**Table S1.** The results of the multicollinearity analysis based on 28 candidate variables.

| Variables | VIF |
| --- | --- |
| sex | 2.127 |
| age | 1.251 |
| LOS | 2.399 |
| PSH | 1.052 |
| smoking history | 2.270 |
| drinking history | 1.621 |
| EDVs | 1.084 |
| ACCI | 2.467 |
| hypertension | 1.105 |
| CHD | 1.086 |
| CI | 1.098 |
| hyperlipidemia | 1.185 |
| chronic gastritis | 1.033 |
| osteoporosis | 1.109 |
| pulmonary infections | 1.112 |
| TC | 1.099 |
| TGs | 3.339 |
| CREA | 2.161 |
| UA | 1.373 |
| LDL-C | 3.002 |
| HDL-C | 1.947 |
| HbA1c | 1.264 |
| FG | 1.330 |
| eGFR | 2.475 |
| NPAR | 1.542 |
| antihypertensive drug use | 1.132 |
| statin use | 1.247 |
| antiplatelet and anticoagulant use | 1.223 |

*PSH: past surgical history; EDVs: emergency department visits; LOS: length of stay; ACCI: age-adjusted Charlson comorbidity index; CHD: coronary heart disease; CI: cerebral infarction; TC:total cholesterol; TGs: triglycerides; CREA: creatinine; UA:uric acid; LDL-C: low density lipoprotein cholesterol; HDL-C: high density lipoprotein cholesterol; HbA1c: glycated hemoglobin; FG:fasting glucose; eGFR: estimated glomerular filtration rate; NPAR: neutrophil percentage-to-albumin ratio; VIF: variance inflation factor.*

**Table S2.** Comparison of continuous variables in the derivation and internal validation cohorts before and after multiple imputation.

|  | Missing variables | Number (%) with missing data | Before  interpolation | After  interpolation | *P* values |
| --- | --- | --- | --- | --- | --- |
| **derivation cohort** | |  |  |  |  |
|  | TC (IQR, mmol/l) | 84(8.80) | 4.24(3.49,5.05) | 4.25(3.49,5.03) | 0.976 |
|  | TGs (IQR, mmol/l) | 84(8.80) | 1.38(1.01,2.05) | 1.37(0.99,2.02) | 0.679 |
|  | CREA (IQR, umol/l) | 200(20.94) | 78.90(63.90,101.95) | 79.30(64.20,101.45) | 0.879 |
|  | UA (IQR, umol/l) | 200(20.94) | 351.00(287.65,428.25) | 348.20(285.25,426.46) | 0.729 |
|  | LDL-C (IQR, mmol/l) | 90(9.42) | 2.34(1.79,3.06) | 2.34(1.78,3.03) | 0.980 |
|  | HDL-C (IQR, mmol/l) | 89(9.32) | 1.08(0.91,1.27) | 1.07(0.91,1.27) | 0.968 |
|  | HbA1c (IQR, %) | 173(18.12) | 7.20(6.50,8.48) | 7.20(6.40,8.30) | 0.488 |
|  | FG (IQR, mmol/l) | 199(20.84) | 7.66(6.00,10.41) | 7.66(5.97,10.72) | 0.492 |
|  | GFR (IQR, ml/min) | 200(20.94) | 75.46(53.36,93.43) | 75.98(53.69,93.43) | 0.778 |
|  | NPAR (IQR, ml/g) | 230(24.08) | 17.86(15.51,20.34) | 17.69(15.37,20.36) | 0.615 |
| **internal validation cohort** | |  |  |  |  |
|  | TC (IQR, mmol/l) | 34(8.31) | 4.28(3.61,5.08) | 4.28(3.60,5.07) | 0.895 |
|  | TGs (IQR, mmol/l) | 34(8.31) | 1.43(1.02,1.94) | 1.43(1.02,1.94) | 0.985 |
|  | CREA (IQR, umol/l) | 78(19.07) | 76.40(60.3,99.90) | 76.30(60.10,98.50) | 0.824 |
|  | UA (IQR, umol/l) | 79(19.32) | 351.05(283.93,413.32) | 352.00(282.90,411.90) | 0.975 |
|  | LDL-C (IQR, mmol/l) | 40(9.78) | 2.36(1.81,3.06) | 2.37(1.80,3.05) | 0.819 |
|  | HDL-C (IQR, mmol/l) | 40(9.78) | 1.09(0.93,1.32) | 1.09(0.93,1.33) | 0.873 |
|  | HbA1c (IQR, %) | 78(19.07) | 7.30(6.58,8.60) | 7.30(6.50,8.60) | 0.878 |
|  | FG (IQR, mmol/l) | 86(21.03) | 7.52(6.05,10.32) | 7.65(6.01,10.40) | 0.533 |
|  | GFR (IQR, ml/min) | 78(19.07) | 76.85(56.36,95.66) | 77.03(57.07,98.51) | 0.727 |
|  | NPAR (IQR, ml/g) | 90(22.00) | 17.36(15.33,19.82) | 17.25(15.29,19.77) | 0.768 |

*TC:total cholesterol; TGs: triglycerides; CREA: creatinine; UA:uric acid; LDL-C: low density lipoprotein cholesterol; HDL-C: high density lipoprotein cholesterol; HbA1c: glycated hemoglobin; FG:fasting glucose; eGFR: estimated glomerular filtration rate; NPAR: neutrophil percentage-to-albumin ratio; IQR: interquartile range.*

**Table S3.** Information of 5 institutions in This Study.

| Institutions | NO.of patients enrolled | No.of 30-day readmission | 30-day readmission rate(%) |
| --- | --- | --- | --- |
| Chongqing Southeast Hospital | 79 | 13 | 16.46 |
| Second Affiliated Hospital of Chongqing Medical University | 889 | 102 | 11.47 |
| University-Town Hospital of Chongqing Medical University | 114 | 13 | 11.40 |
| Third Affiliated Hospital of Chongqing Medical University | 282 | 41 | 14.54 |
| Affiliated Banan Hospital of Chongqing Medical University | 535 | 79 | 14.77 |
| Total | 1899 | 248 | 13.06 |

**Table S4.** Distribution of anti-hypertensive drug types.

| Anti-hypertensive drug types | No.of anti-hypertensive drug use | No.of 30-day readmission | 30-day readmission rate (%) |
| --- | --- | --- | --- |
| diuretics | 251 | 48 | 19.12 |
| β blockers | 161 | 21 | 13.04 |
| angiotensin receptor blockers | 111 | 14 | 12.61 |
| α blockers | 8 | 1 | 12.50 |
| calcium channel blockers | 226 | 27 | 11.95 |
| fixed-dose combination | 58 | 6 | 10.34 |
| angiotensin-converting enzyme inhibitors | 44 | 4 | 9.09 |
| α+β blockers | 3 | 0 | 0 |
| absence of antihypertensive medications | 93 | 3 | 3.23 |
| total | 955 | 124 | 12.98 |

**Table S5.** Results of multicollinearity analysis

| Variables | VIF |
| --- | --- |
| age | 1.137 |
| PSH | 1.012 |
| osteoporosis | 1.028 |
| pulmonary infections | 1.091 |
| antihypertensive drug use | 1.021 |
| CREA | 1.904 |
| eGFR | 2.022 |
| NPAR | 1.138 |

*PSH: past surgical history; CREA: creatinine; eGFR: estimated glomerular filtration rate; NPAR: neutrophil percentage-to-albumin ratio; VIF: variance inflation factor.*

**Table S6.** The AUROC values for different numbers of predictor variables.

| Number of Predictors | Ensemble Predictors | AUROC (95%CI) | P value |
| --- | --- | --- | --- |
| 1 | pulmonary infections | 0.631 (0.588-0.674) | / |
| 2 | pulmonary infections+NPAR | 0.765 (0.716-0.813) | <0.001 |
| 3 | pulmonary infections+NPAR+antihypertensive drug use | 0.782 (0.737-0.826) | <0.001 |
| 4 | pulmonary infections+NPAR+antihypertensive drug use+PSH | 0.780 (0.736-0.824) | 0.765 |
| 5 | pulmonary infections+NPAR+antihypertensive drug use+PSH+eGFR | 0.777 (0.730-0.821) | 0.330 |
| 6 | pulmonary infections+NPAR+antihypertensive drug use+PSH+eGFR+OP | 0.777 (0.733-0.822) | 0.754 |
| 7 | pulmonary infections+NPAR+antihypertensive drug use+PSH+eGFR+OP+age | 0.777 (0.733-0.823) | 0.982 |
| 8 | pulmonary infections+NPAR+antihypertensive drug use+PSH+eGFR+OP+age+CREA | 0.778 (0.733-0.823) | 0.526 |

*NPAR: neutrophil percentage-to-albumin ratio; PSH: past surgical history; eGFR: estimated glomerular filtration rate; CREA: creatinine; OP: osteoporosis; AUROC: area under the receiver operating characteristic curve; CI: Confidence Interval.*

**Figure S1.** Flow of inclusions and exclusions.

**
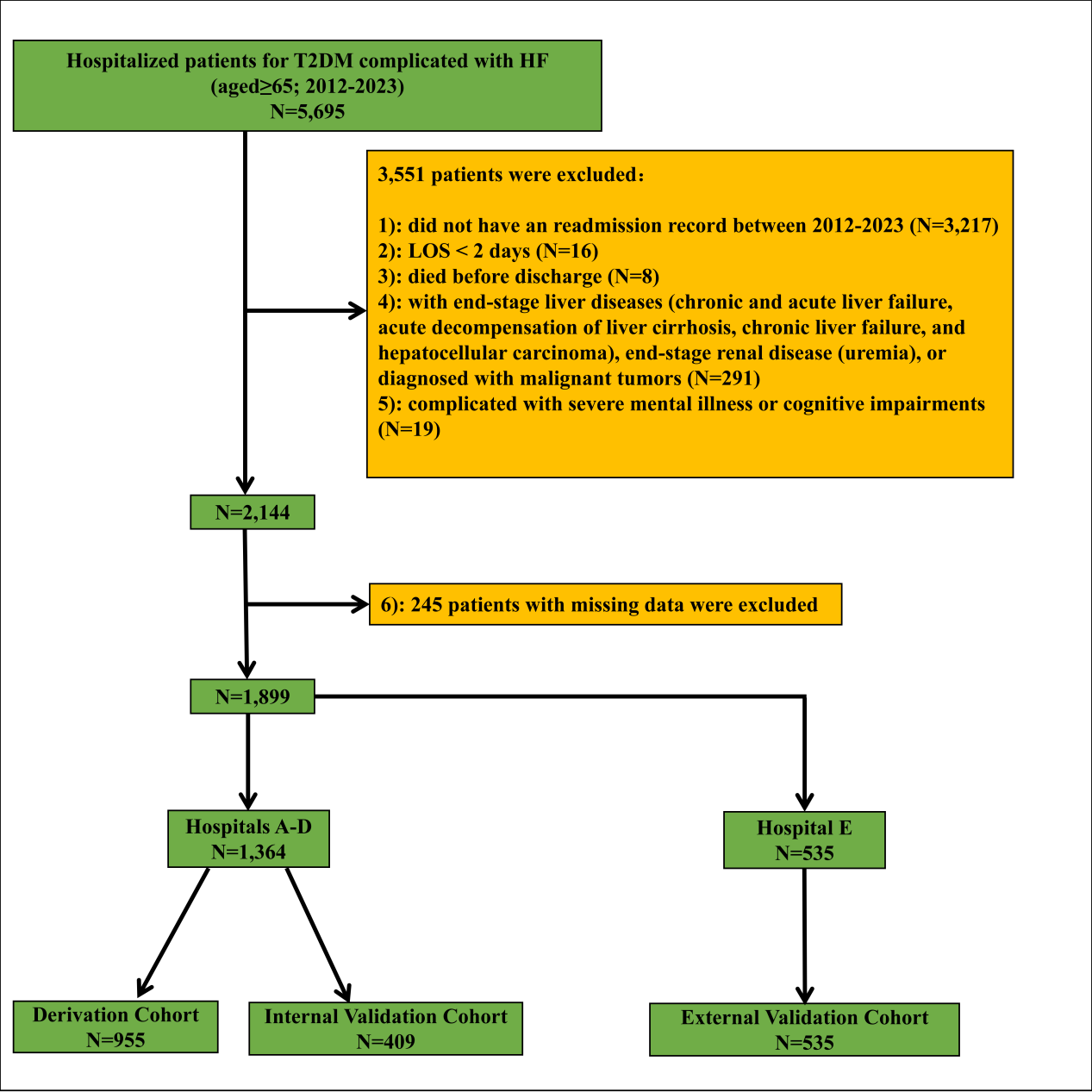
**

**Figure S2.** The distribution and relationships of individual variables with 30-day readmission.


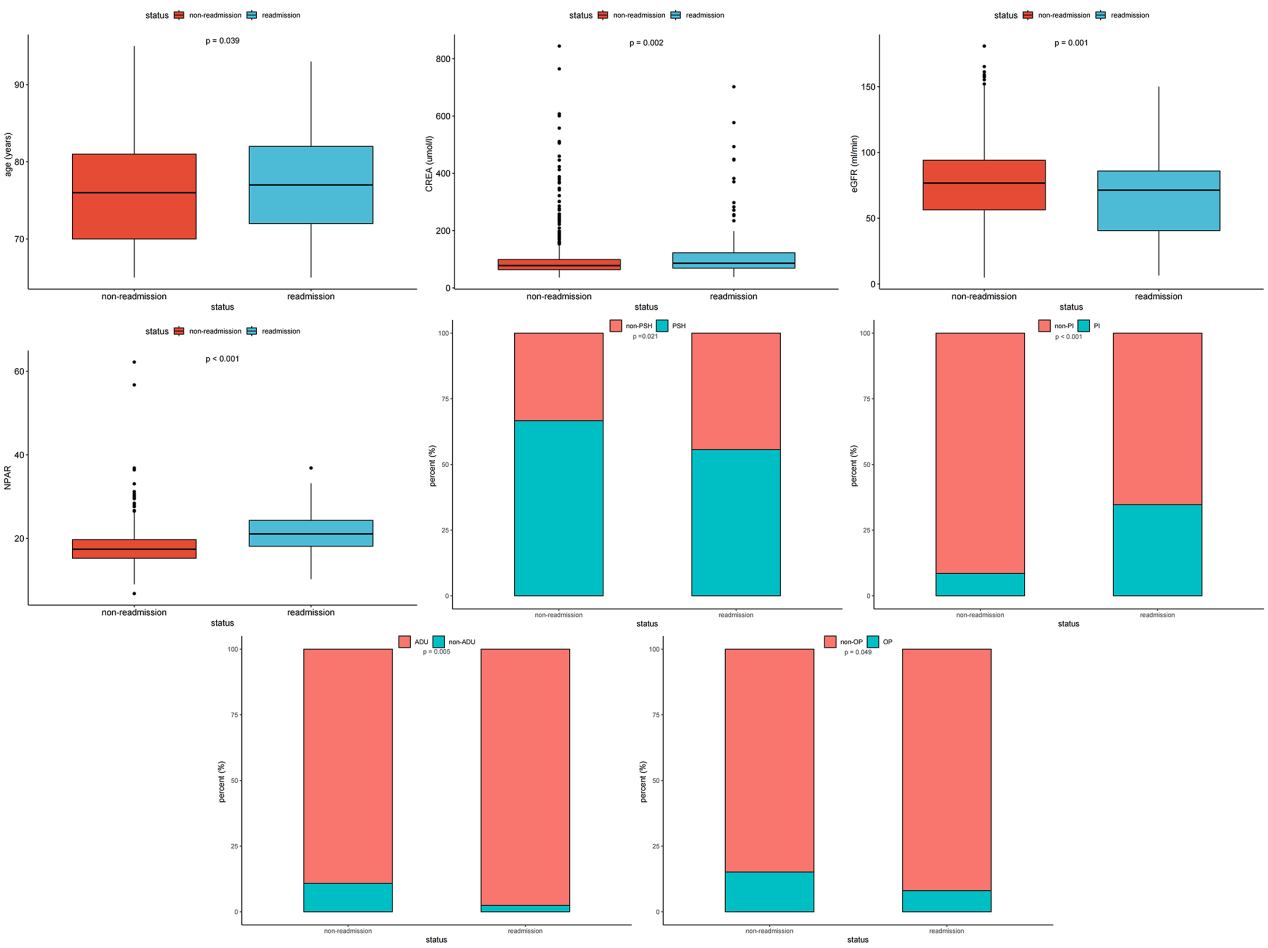


**Figure S3.** The results of correlation matrix.


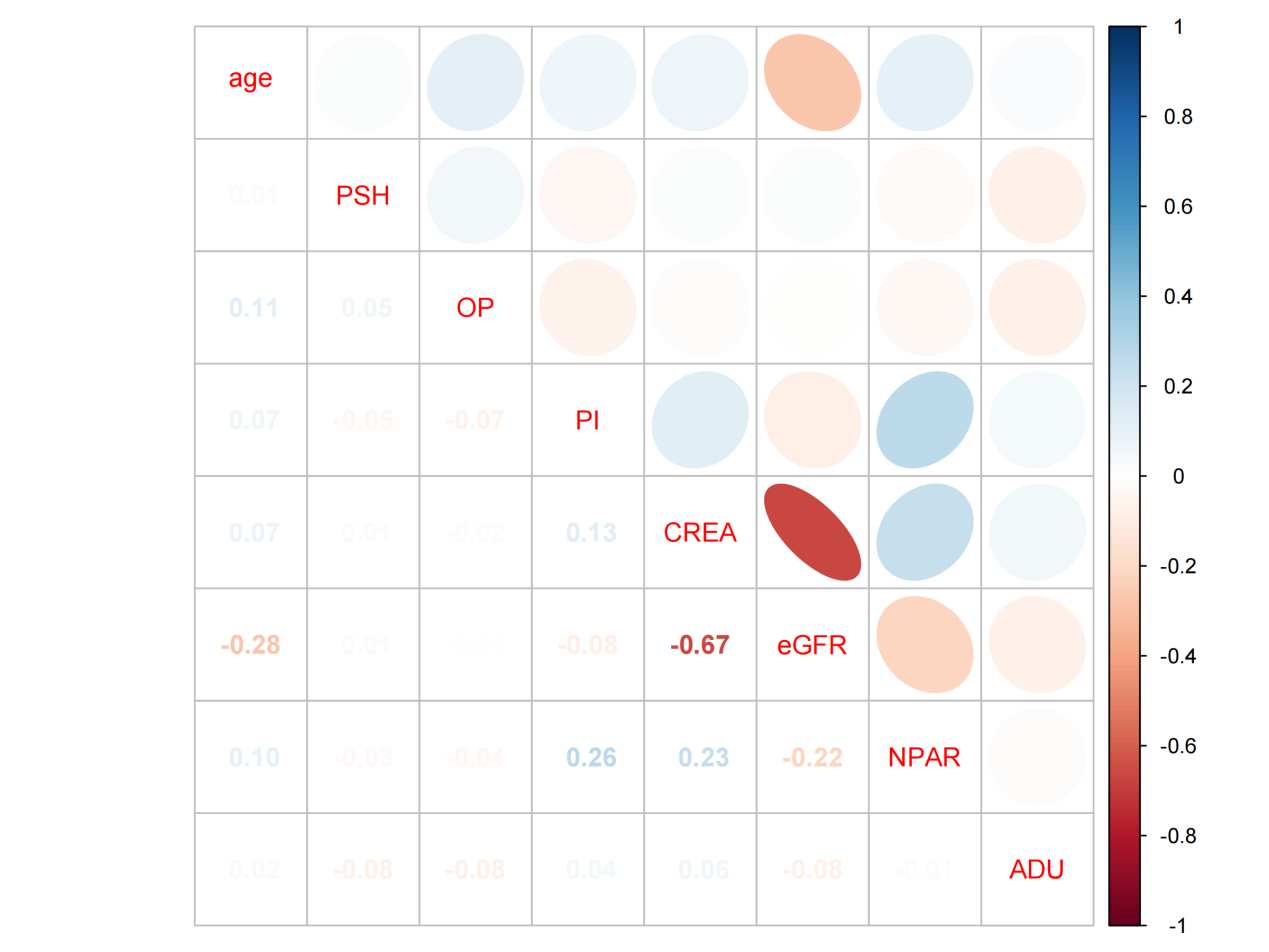


**Figure S4.** Identification of the optimal variables numbers for a prediction of 30-day readmission.

**
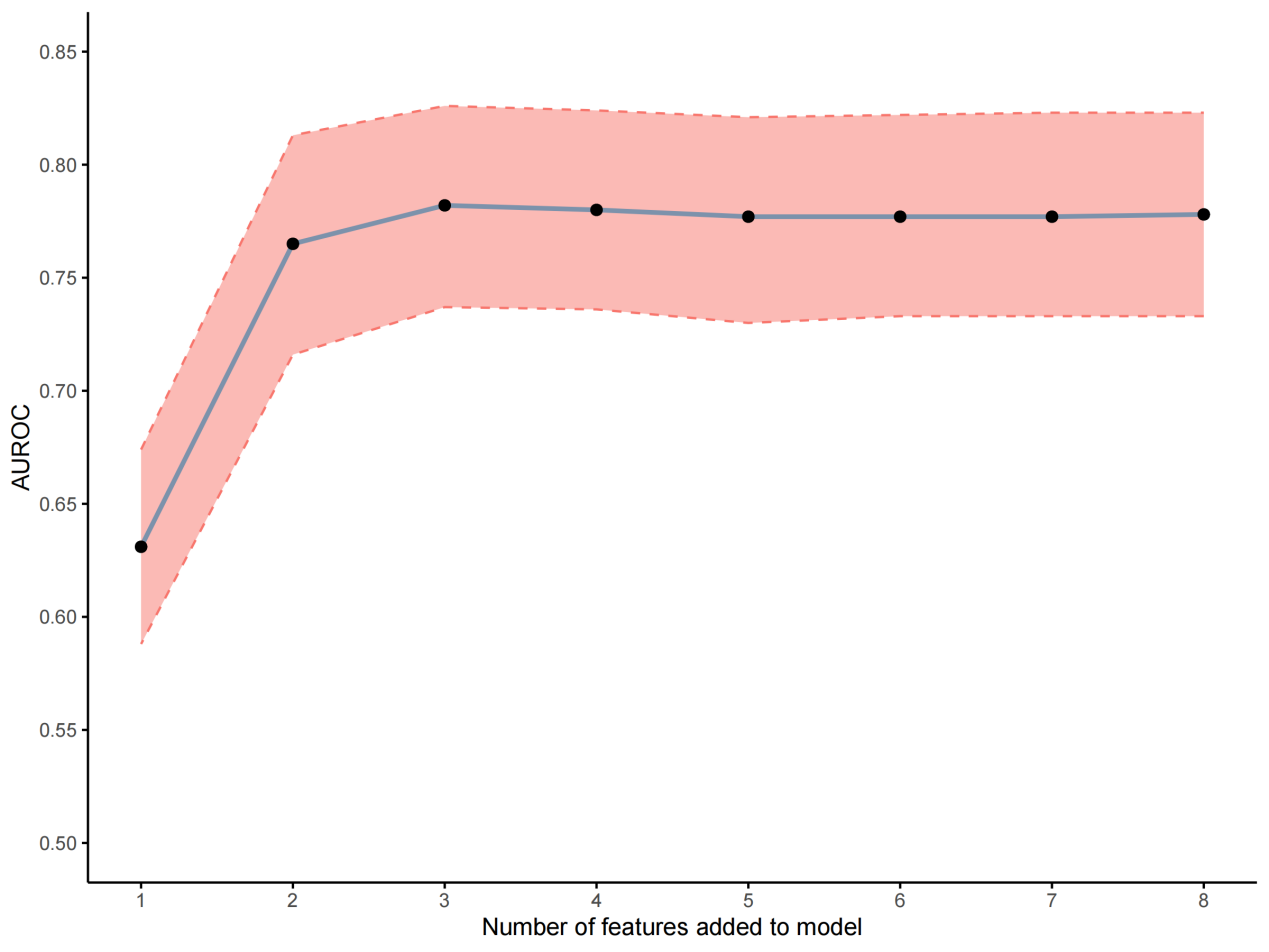
**

**Figure S5.** Calibration curve of the model in the internal validation cohort. The x-axis represents the predicted probability of 30-day readmission. The y-axis represents the actual occurred 30-day readmission. the black dashed line represents the perfect prediction with the same predicted probability as the actual probability. The blue dashed line represents the performance of the nomogram and the red solid line represents the performance of the model after calibration. The closer the calibration curve of the model is to the black dashed line, the better the model prediction is represented.

**
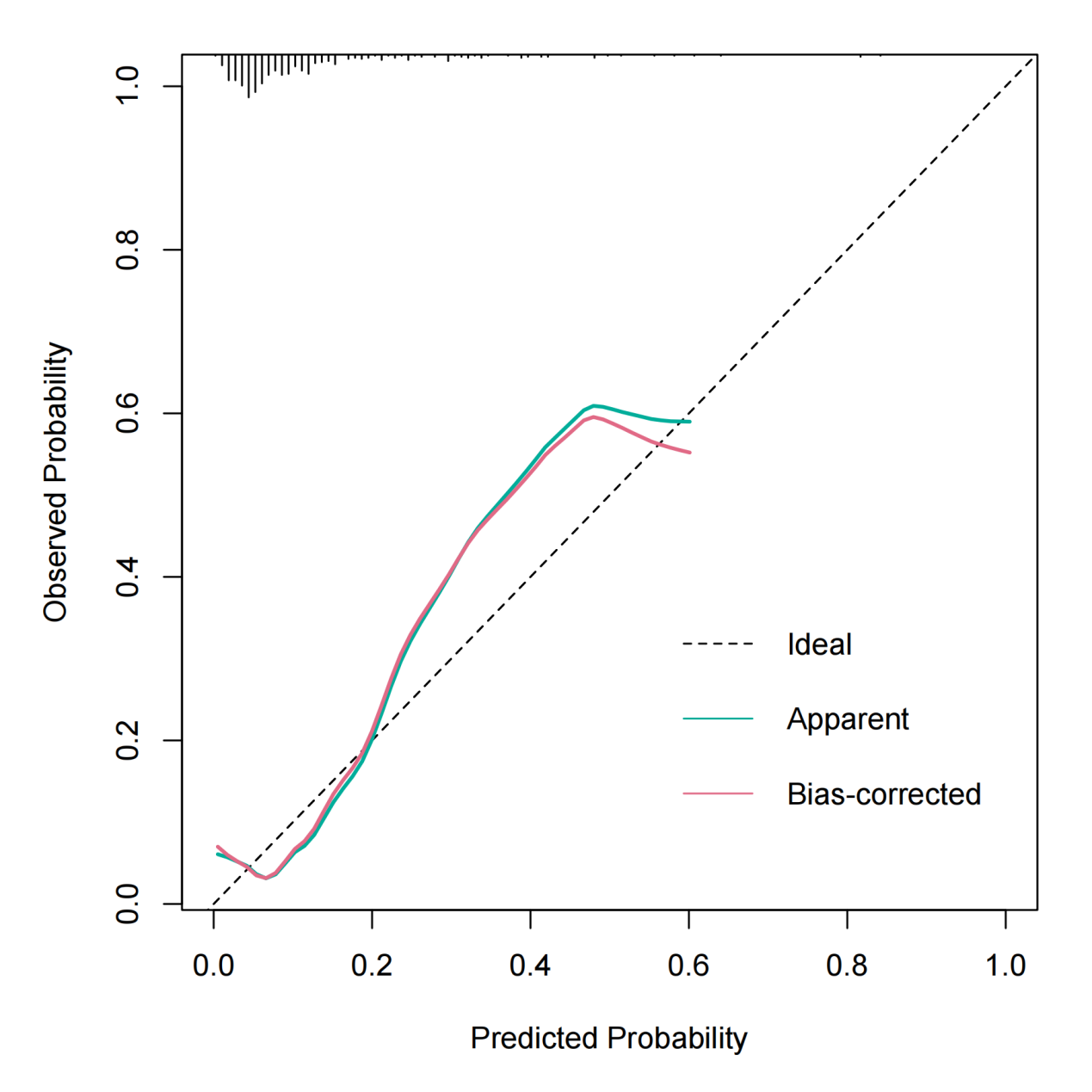
**

**Figure S6.** Calibration curve of the model in the external validation cohort. The x-axis represents the predicted probability of 30-day readmission. The y-axis represents the actual occurred 30-day readmission. the black dashed line represents the perfect prediction with the same predicted probability as the actual probability. The blue dashed line represents the performance of the nomogram and the red solid line represents the performance of the model after calibration. The closer the calibration curve of the model is to the black dashed line, the better the model prediction is represented.

**
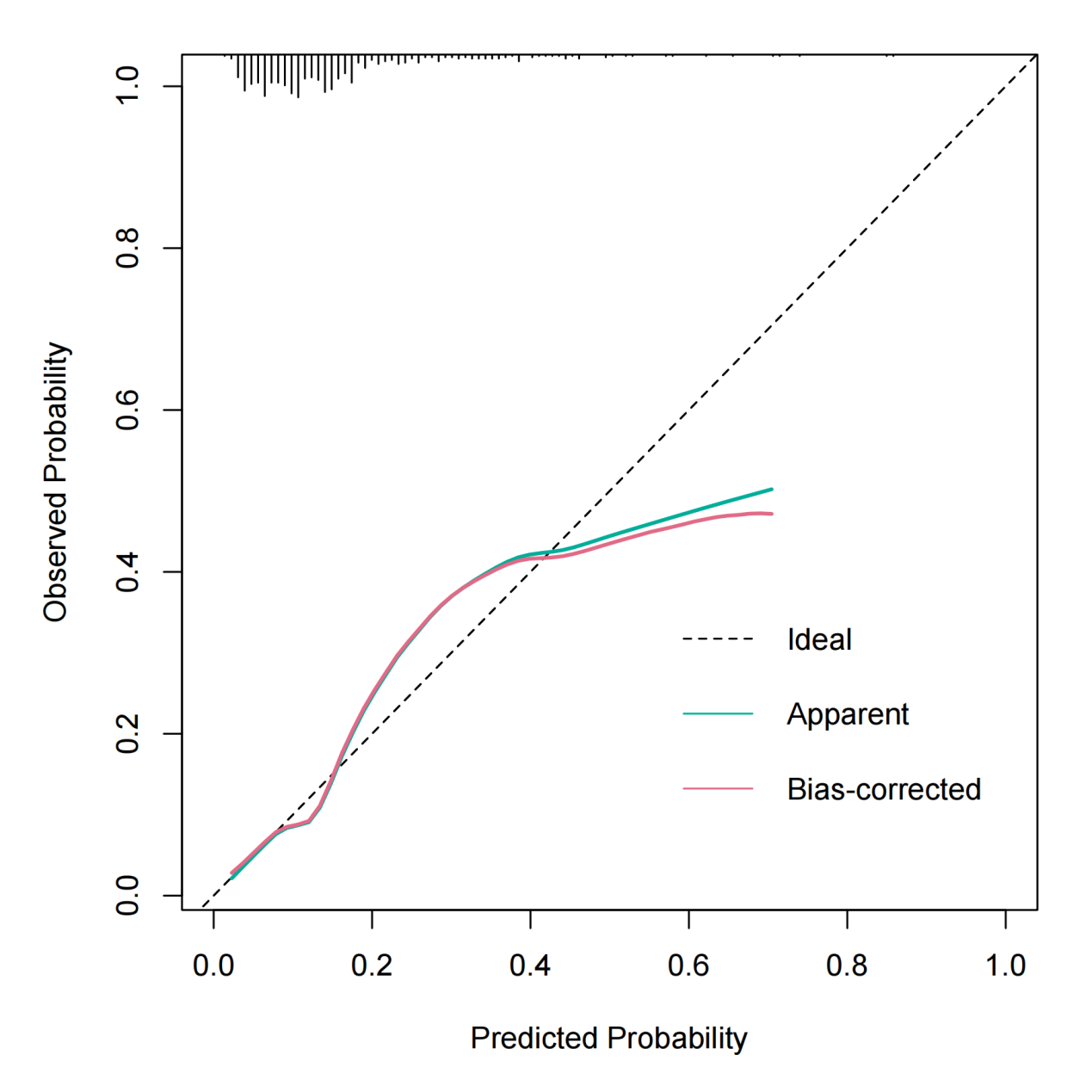
**

**Figure S7.** DCA of the model in the internal validation cohort. The net benefits were measured at different threshold probabilities. The gray line represents the assumption that all patients are identified as 30-day readmission. The black line represents the assumption that no patients are identified as 30-day readmission.

**
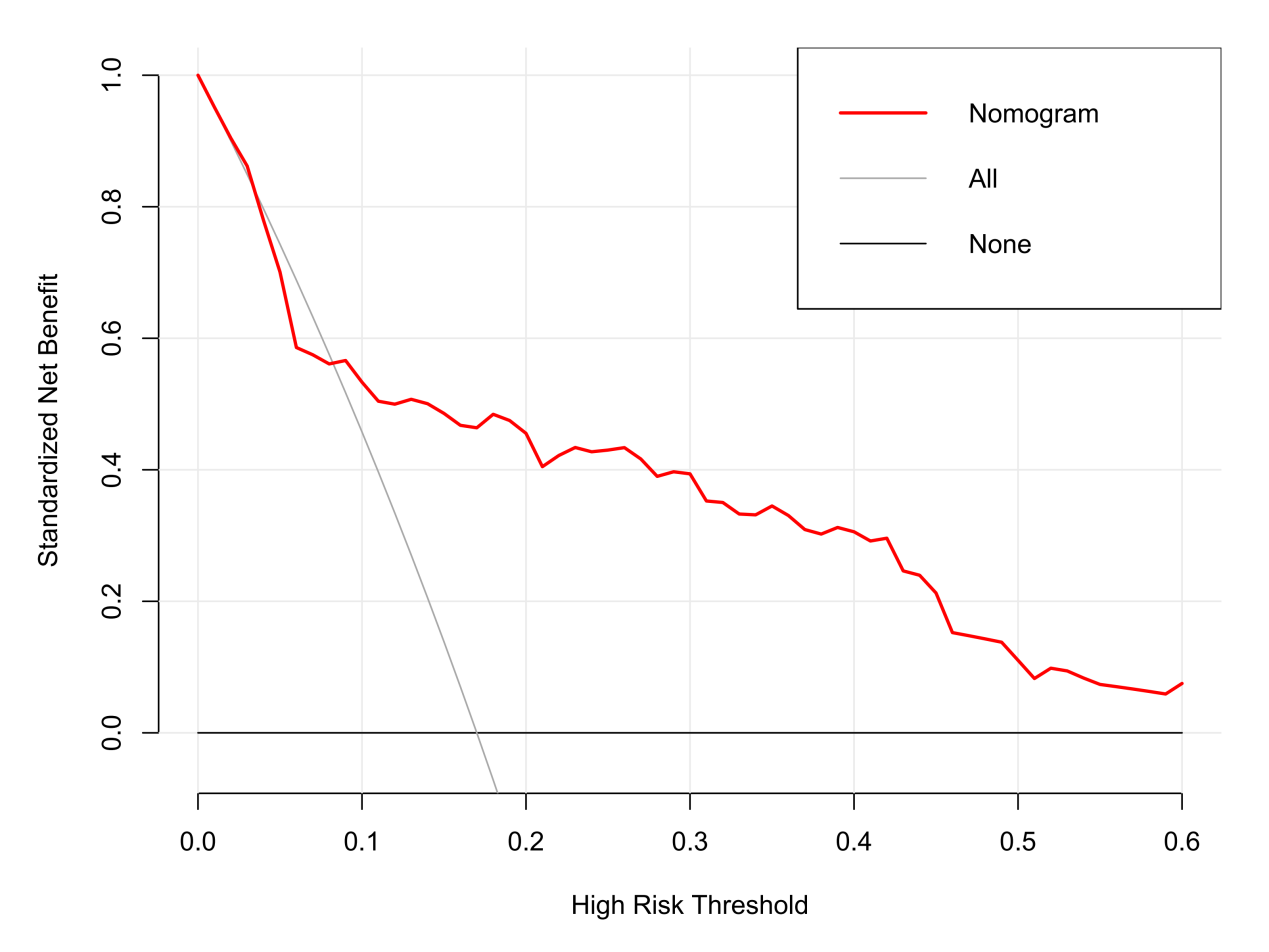
**

**Figure S8.** DCA of the model in the external validation cohort. The net benefits were measured at different threshold probabilities. The gray line represents the assumption that all patients are identified as 30-day readmission. The black line represents the assumption that no patients are identified as 30-day readmission.

**
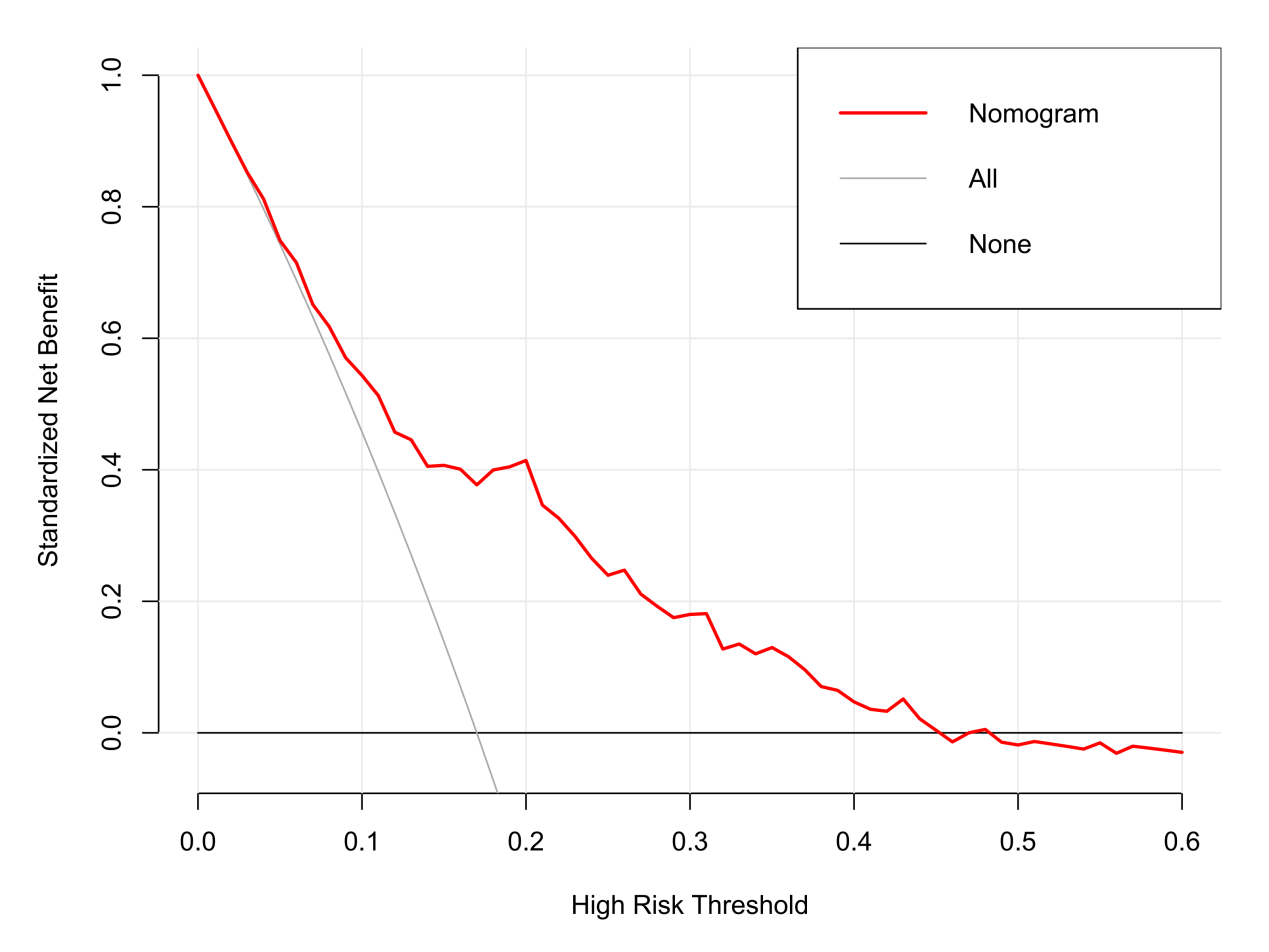
**

**Figure S9.** CIC of the model in the internal validation cohort. The clinical impact curve illustrated the number of 30-day readmission in a sample population of 1000. The blue curve indicates the predicted number of 30-day readmission at various threshold probabilities, while the red curve represents the actual number of 30-day readmission.

**
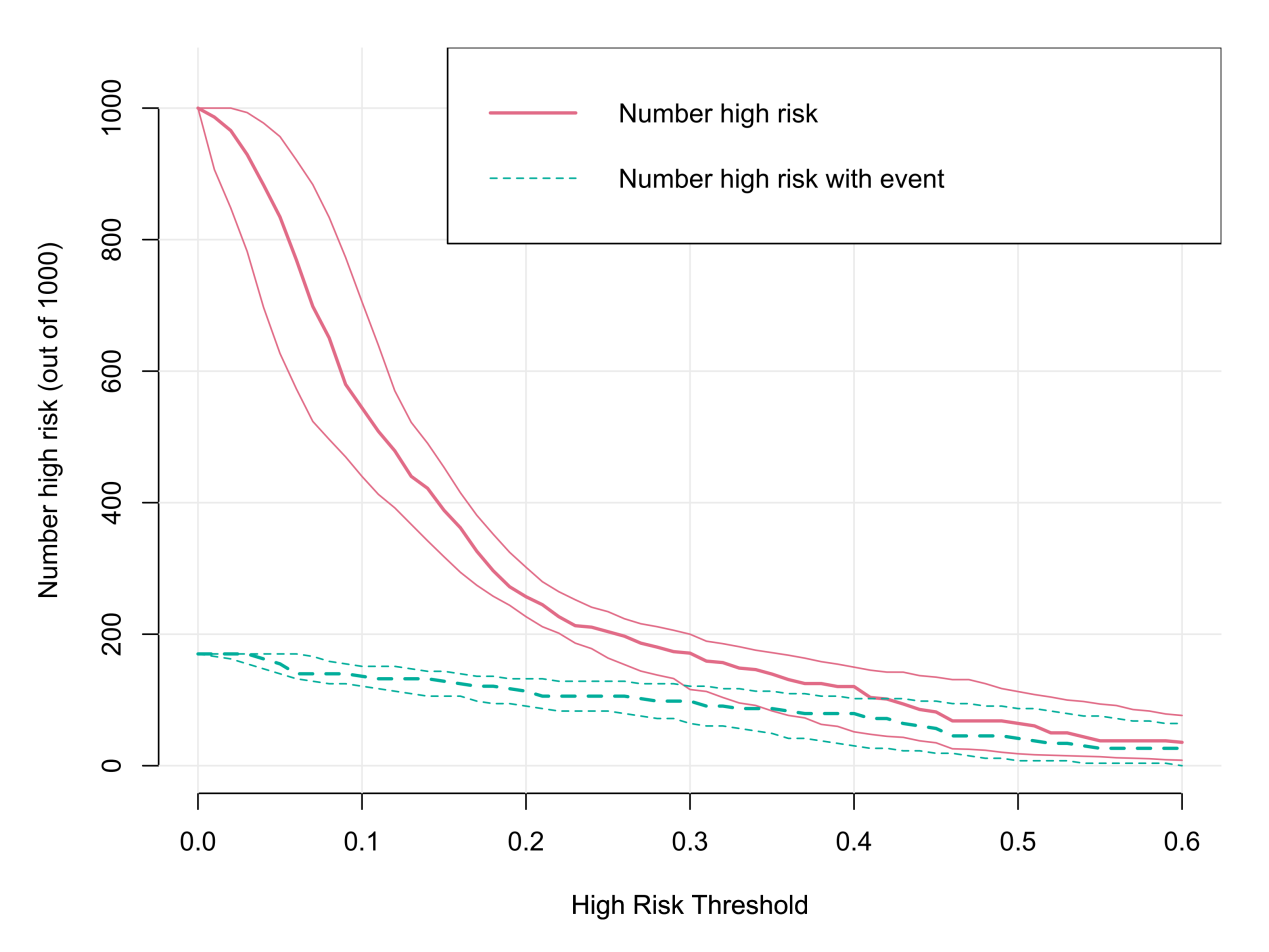
**

**Figure S10.** CIC of the model in the external validation cohort. The clinical impact curve illustrated the number of 30-day readmission in a sample population of 1000. The blue curve indicates the predicted number of 30-day readmission at various threshold probabilities, while the red curve represents the actual number of 30-day readmission.

**
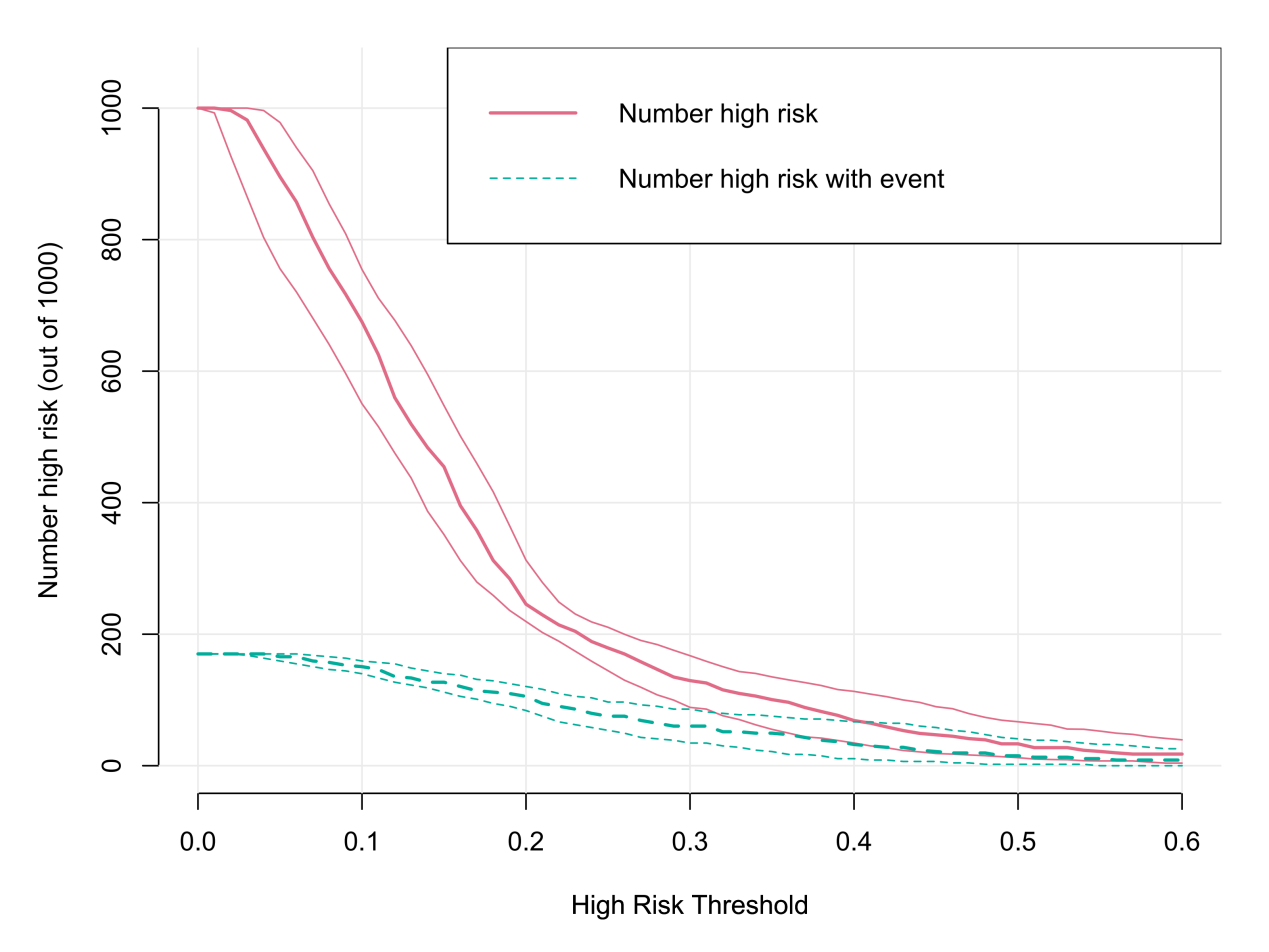
**
